# Extracellular molecular signals shaping dendrite architecture during brain development

**DOI:** 10.3389/fcell.2023.1254589

**Published:** 2023-12-07

**Authors:** Mohammad I. K. Hamad, Bright Starling Emerald, Kukkala K. Kumar, Marwa F. Ibrahim, Bassam R. Ali, Mo’ath F. Bataineh

**Affiliations:** ^1^ Department of Anatomy, College of Medicine and Health Sciences, United Arab Emirates University, Al Ain, United Arab Emirates; ^2^ Department of Genetics and Genomics, United Arab Emirates University, Al Ain, United Arab Emirates; ^3^ Department of Nutrition and Health, College of Medicine and Health Sciences, United Arab Emirates University, Al Ain, United Arab Emirates

**Keywords:** dendritic development, neurotransmitters, extracellular matrix, neurotrophins, neuregulins, cadherins and protocadherins, ephrin, reelin

## Abstract

Proper growth and branching of dendrites are crucial for adequate central nervous system (CNS) functioning. The neuronal dendritic geometry determines the mode and quality of information processing. Any defects in dendrite development will disrupt neuronal circuit formation, affecting brain function. Besides cell-intrinsic programmes, extrinsic factors regulate various aspects of dendritic development. Among these extrinsic factors are extracellular molecular signals which can shape the dendrite architecture during early development. This review will focus on extrinsic factors regulating dendritic growth during early neuronal development, including neurotransmitters, neurotrophins, extracellular matrix proteins, contact-mediated ligands, and secreted and diffusible cues. How these extracellular molecular signals contribute to dendritic growth has been investigated in developing nervous systems using different species, different areas within the CNS, and different neuronal types. The response of the dendritic tree to these extracellular molecular signals can result in growth-promoting or growth-limiting effects, and it depends on the receptor subtype, receptor quantity, receptor efficiency, the animal model used, the developmental time windows, and finally, the targeted signal cascade. This article reviews our current understanding of the role of various extracellular signals in the establishment of the architecture of the dendrites.

## 1 Introduction

**FIGURE 1:**
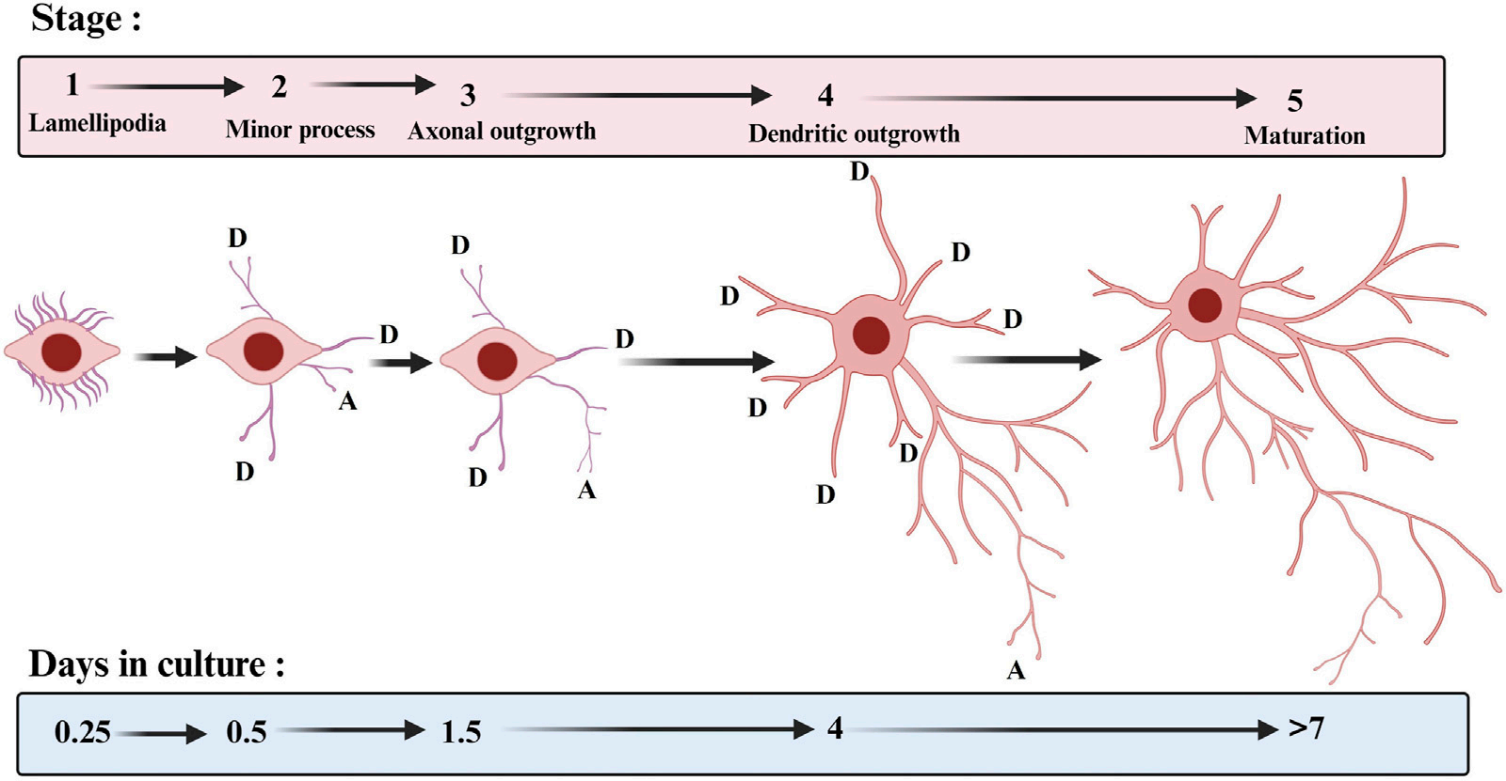
Stages of hippocampal neuronal development in culture. The approximate times when cells enter each of the stages are indicated. D: dendrite and A: axon. The template for this figure was (Dotti et al., 1988).

The dendrites of neurons represent the main input compartment, and the proper growth and arborisation of dendrites are crucial for the proper functioning of the central nervous system. The dendrites of a neuron contain a variety of receptors designed to receive signal input from other cells for communication, differentiation, and maturation. In neurons, axon generation (axogenesis) and specification precede dendritogenesis (Barnes and Polleux, 2009). The neurites are specified to become axon or dendrites. Generally, dendrites differ from axons at the molecular, functional, and morphological levels. The initial polarization of the neuron is regulated by both extrinsic factors and intrinsic cues (see review by Horton and Ehlers, 2003). *In vitro* and *in vivo* studies have shown that few nascent processes extend from the soma, and one of these will differentiate into the axon and grow out of the soma, whereas the remaining neurites are destined to become dendrites (Dotti and Banker, 1987; Dotti et al., 1988) (Figure 1). Most studies on dendritic growth have shown that dendritic growth process is extremely dynamic (Cline, 2001; Prigge and Kay, 2018). The new dendritic branches extend and retract undergoing constant remodeling. In most neuronal types, dendrites grow away from the cell body using guidance molecules, so they increase in length and diameter and, thereafter, they start to bifurcate to increase their complexity. Both extrinsic and intrinsic factors are involved in dendritic growth process but for the scope of this review we will focus only on extrinsic factors that regulate dendritic growth. There are mechanisms that ensure that dendritic arbors maximise their spread across a territory while minimising the redundancy of the innervated area between dendrites of different neurons, a process known as dendritic tilling (Grueber and Sagasti, 2010). Meanwhile, dendrites develop specialized structures named dendritic spines (spinogenesis), which are one of the important contact sites for communication between neurons to ensure proper network maturation (Ben-Ari, 2001). At this point, neurons refine and modify their dendritic tree through retraction and elimination of dendritic parts (dendritic pruning). It is now well recognized that pruning of dendrites as a mean to refine neuronal networks is a widespread phenomenon required for the normal development of vertebrate and invertebrate nervous systems (for a review, see Schuldiner and Yaron, 2015).

During early development, dendritic growth is regulated by both cell-intrinsic programmes and extrinsic factors that regulate various aspects of dendritic development (Jan and Jan, 2003; Lin et al., 2020). It was always believed that dendritic growth is intrinsically determined. However, in the past 2 decades, numerous studies have shown that the dendritic growth process is remarkably responsive to extrinsic factors, influencing local and global mechanisms of dendrite development (Valnegri et al., 2015). The extrinsic factors include neurotransmitters, neurotrophins, extracellular matrix proteins, contact-mediated ligands, and secreted and diffusible cues. On the other hand, the intrinsic factors that play a key role in dendritic development are mediated by specific transcription factors. They play a key role in determining the neuronal identity, morphological features, positioning and functional electrical properties of the neuronal cells; comprehensive reviews on the role of the transcription factor in dendritic growth development appear elsewhere (McAllister, 2000; Jan and Jan, 2003; Schuldiner and Yaron, 2015; Ledda and Paratcha, 2017). The neuronal membrane is involved in neuronal preservation and functioning. For a neuron to respond to an external stimulus, it is necessary to have sufficient active membrane receptors. Therefore, manipulating the neuronal receptor quantity or efficiency can affect the dendritic growth process. The Ca^2+^ set-point hypothesis for dendritic growth suggests that too little or too much intracellular Ca^2+^ inhibits neurite growth, whereas maintaining a moderate intracellular Ca^2+^ level is essential for proper dendritic development (Lohmann and Wong, 2005). Ca^2+^ signaling impacts dendritic growth by triggering specific signalling cascades. In addition, Ca^2+^ signalling influences dendrite dynamics through modulation of the cytoskeleton near the Ca^2+^ entry site, whereas Ca^2+−^dependent dendritic growth involves activating specific transcription factors (Konur and Ghosh, 2005). Ca^2+^ signalling through the intracellular CaMKII also has been implicated in dendritic growth in early development. CaMKII has two different isoforms, CaMKIIα which restrict dendritic growth (Wu and Cline, 1998; Redmond et al., 2002) and CaMKIIβ which promote dendritic growth (Fink et al., 2003), suggesting that CaMKs are receptor downstream Ca^2+−^dependent dendritic growth regulators.

Neuronal activity has been shown to shape synaptic connectivity early in the development of the central nervous system (Katz and Shatz, 1996; Kirischuk et al., 2017). One remarkable feature of the developing brain is the appearance of spontaneous neuronal activity (Khazipov and Luhmann, 2006; Luhmann et al., 2016). The early network spontaneous activity patterns coincide with the period of rapid dendritic growth dependent on Ca^2+^ signalling (Ben-Ari, 2001). Blocking the synaptic activity in optic tectum neurons delayed dendritic development *in vivo* (Rajan and Cline, 1998). Enhanced visual activity driven by a light stimulus has been shown to promote dendritic growth (Sin et al., 2002). Visual deprivation from dark rearing in the visual system decreases the dendritic growth of stellate neurons (Coleman and Riesen, 1968). In addition, the overexpression or the elimination of certain receptors that increase neuronal excitability affects the dendritic growth process (Haas et al., 2006; Hamad et al., 2011; Jack et al., 2019). Therefore, neuronal activity is key in dendritic growth during early development. This review summarises and critically evaluates the extracellular molecular signals that control dendrite architecture. Additionally, we present our perspectives on the implications of abnormal dendritic morphology for nervous system diseases.

## 2 Extracellular molecular signals regulating dendritic growth: Classes and mechanisms of action

### 2.1 Neurotransmitters

#### 2.1.1 Glutamate

**FIGURE 2:**
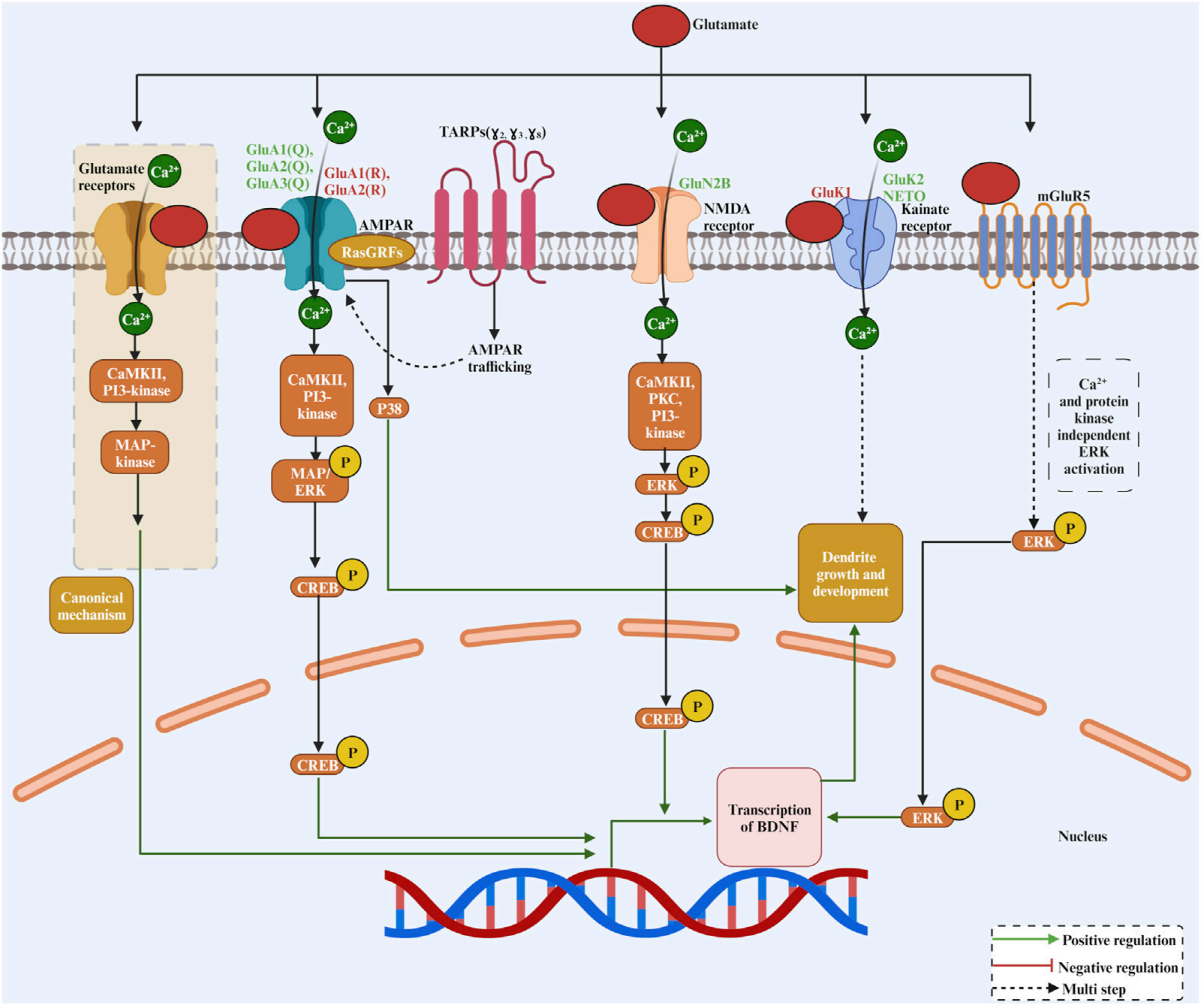
The complex role of glutamate as a neurotransmitter and its receptors in dendritic growth. Glutamate binds to a variety of receptors, including AMPAR, NMDA, Kainate and mGluR5, activating CREB in a Ca^2+^ and kinase-dependent manner except for mGluR5 for transcriptional activation of genes required for dendritic growth and development.

Glutamate is a major neurotransmitter that regulates dendritic growth, and glutamatergic transmission convergence with Ca^2+^ signalling plays a key role in dendritic differentiation. Based on pharmacology and structural homology, the Glutamate receptors are grouped into four distinct classes, including the AMPA receptors (GluA1–GluA4), the kainate receptors (GluK1–GluK5), the NMDA receptors (GluN1, GluN2A–GluN2D, GluN3A, and GluN3B), and the δ receptors (GluD1 and GluD2) (Traynelis et al., 2010). Among these classes, the AMPAR-type glutamate receptor (AMPAR) mediates the fastest excitatory synaptic transmission in the brain. Studies investigating the role of glutamate and its receptors in dendritic growth are summarised in Table 1. The efficacy of AMPARs in enhancing dendritic growth depends on the overexpressed subunit editing site and splice variant. Regarding the editing variants, the overexpression of the Ca^2+^-permeable AMPAR subunit containing the unedited (Q) variant was reported to enhance dendritic growth (Inglis et al., 2002; Jeong et al., 2006; Prithviraj et al., 2008; Hamad et al., 2011). On the other hand, the overexpression of the edited Ca^2+^-impermeable variant (R) frequently did not affect dendritic growth (Inglis et al., 2002; Prithviraj et al., 2008; Hamad et al., 2011) (Figure 2). The presence of the edited variant (R) in heteromeric AMPARs renders the channel impermeable to Ca^2+^ and influences channel kinetics and single-channel conductance, whereas the Ca^2+^-permeable variant (Q) renders the channel permeable to Ca2+ (Burnashev et al., 1992). Thus, the Ca^2+^ permeability of the overexpressed subunit is a determining factor in promoting dendrite growth. Splice variants of AMPARs can also control dendritic growth. All four AMPAR subunits occur in two spliced versions, named flip-and-flop. The flip-flop change occurs in the proximity of the ligand-binding domain in the extracellular loop (Sommer et al., 1990). In neurons, flip variants are usually predominant in early developmental stages and are replaced by flop variants in adulthood (Monyer et al., 1991). The flip variants have slower desensitisation time than the flop variant, keeping the channel open longer, and thereby allowing more Ca^2+^ influx into the cell. Expectedly, overexpression of the AMPAR subunits GluA1, GluA2, and GluA3 harbouring the flip variant improved dendritic growth (Inglis et al., 2002; Jeong et al., 2006; Prithviraj et al., 2008; Hamad et al., 2011), while overexpression of the flop variant did not affect dendritic growth in neocortical neurons (Hamad et al., 2011). On the other hand, the overexpression of AMPARs GluA1 and GluA2 C-terminal domains in optic tectal neurons reduced dendritic growth (Haas et al., 2006). However, in P28, the overexpression of the GluA1 subunit did not alter the dendritic length (Jeong et al., 2006). Generally, blocking glutamate receptors in rat neocortical organotypic cultures reduced the dendritic growth of these neurons (Baker et al., 1997). Furthermore, the stimulation with the positive allosteric modulators of AMPARs (ampakines) increased the dendritic growth of hippocampal pyramidal neurons in adult rats (Lauterborn et al., 2016). Although chronic treatment with ampakines did not disturb basic synaptic physiology, it positively affected long-term potentiation. Taken together, these studies suggest that AMPARs play a key role in dendritic development and such effect relies on these receptors editing and splice variants. Generally, the Ca^2+^ permeability of AMPARs is a key factor for promoting dendritic growth.

**TABLE 1:** Summary of the studies on the role of neurotransmitters and their receptors in dendritic growth. Days *in vitro* (DIV). Postnatal day (P). Embryonic (E).

Protein	Animal and age	System	Strategy	Growth effect	Reference
**AMPARs**					
**AMPARs**	rat 10 months	hippocampal pyramidal cells	activation	increased	Lauterborn et al. (2016)
**AMPARs**	frog stage 46–47	optic tectal neurons	blocking	no change	Rajan and Cline (1998)
**GluA1**	rat 10 DIV	neocortical interneuron	overexpression	increased	Hamad et al. (2011)
**GluA1**	rat P28	cultured motor neurons	overexpression	No change	Jeong et al. (2006)
**GluA1**	rat 8 DIV	dissociated neocortical neurons	overexpression	increased	Chen et al. (2009a)
**GluA1**	rat P28	cultured motor neurons	overexpression	increased	Inglis et al. (2002)
**GluA1**	rat 7 DIV	mixed spinal cord cultures	overexpression	increased	Prithviraj et al. (2008)
**GluA1**	mouse 9 DIV	mixed spinal cord culture	overexpression	increased	Zhou et al. (2008)
**GluA1**	mouse P10-13	motor neurons	knockout	decreased	Zhang et al. (2008)
**GluA1 (CT)**	frog stage 46–47	optic tectal neurons	overexpression	decreased	Haas et al. (2006)
**GluA1**	frog stage 46–47	optic tectal neurons	blocking	decreased	Haas et al. (2006)
**GluA2**	rat 10 DIV	neocortical pyramidal cells	overexpression	increased	Hamad et al. (2011)
**GluA2**	rat 8 DIV	dissociated neocortical neurons	overexpression	increased	Chen et al. (2009a)
**GluA2**	rat 7 DIV	mixed spinal Cord cultures	overexpression	increased	Prithviraj et al. (2008)
**GluA2**	frog stage 46–47	optic tectal neurons	blocking	decreased	Haas et al. (2006)
**GluA2 (CT)**	frog stage 46–47	optic tectal neurons	overexpression	decreased	Haas et al. (2006)
**GluA3**	rat 10 DIV	neocortical pyramidal cells	overexpression	increased	Hamad et al. (2011)
**TARPs**					
**TARPs γ-2**	rat 15 DIV	neocortical pyramidal cells	overexpression	increased	Hamad et al. (2014)
**TARPs γ-3**	rat 15 DIV	neocortical pyramidal cells	overexpression	increased	Hamad et al. (2014)
**TARPs γ-4**	rat 15 DIV	neocortical pyramidal cells	overexpression	decreased	Hamad et al. (2014)
**TARPs γ-5**	rat 15 DIV	neocortical pyramidal cells	overexpression	no change	Hamad et al. (2014)
**TARPs γ-7**	rat 15 DIV	neocortical pyramidal cells	overexpression	no change	Hamad et al. (2014)
**TARPs γ-8**	rat 15 DIV	neocortical pyramidal cells	overexpression	increased	Hamad et al. (2014)
**NMDARs**					
**GluN2A**	rat 10 DIV	cortical pyramidal cells	blocking	no change	Gonda et al. (2020)
**GluN2A**	mouse adult	hippocampal DG neurons	knockout	decreased	Kannangara et al. (2014)
**GluN2B**	rat 10DIV	cortical pyramidal cells	blocking	increased	Gonda et al. (2020)
**GluN2B mutant**	rat 9 DIV	neocortical neuron	overexpression	decrease	Bahry et al. (2021)
**GluN2B mutant**	mouse 10–14 DIV	cortical neurons	overexpression	decrease	Sceniak et al. (2019)
**GluNR1**	mouse P5	trigeminal principal neurons	knockout	increased	Lee et al. (2005)
**NMDAR**	mouse P21	spinal motor neurons	blocking	decreased	Kalb (1994)
**NMDARs**	frog stage 46–47	optic tectal neurons	blocking	decreased	Rajan and Cline (1998)
**NMDARs**	mouse P8	motor neurons	blocking	decreased	Inglis et al. (1998)
**NR2B**	mouse P21	hippocampus, granule cells	knockout	no change	Espinosa et al. (2009)
**NR2B**	mouse P21	cortical spiny stellate	knockout	no change	Espinosa et al. (2009)
**NR3B**	rat 7 DIV	cultured spinal motor neurons	overexpression	increased	Prithviraj and Inglis (2008)
**KARs and auxiliary subunits**					
**GluK1**	rat 10 DIV	neocortical interneurons	overexpression	increased	Jack et al. (2019)
**GluK2**	rat 10 DIV	neocortical pyramidal cells	overexpression	increased	Jack et al. (2019)
**Neto1**	rat 10 DIV	neocortical interneurons	overexpression	increased	Jack et al. (2019)
**Neto2**	adult	dorsal root ganglion neurons	knockout	decreased	Vernon and Swanson (2017)
**δ receptors**					
**GluD2**	mouse P21	Purkinje Cells	knockout	no change	Takeo et al. (2021)
**Metabotropic glutamate receptors**					
**mGluR1**	mouse 5 DIV	cerebellar Purkinje neurons	activation	decreased	Sirzen-Zelenskaya et al. (2006)
**mGluR5**	mouse P6 –P7	neocortical spiny stellate	knockout	increased	Ballester-Rosado et al. (2010)
**Serotonin receptors**					
**5-HT receptors**	mouse E15.5	cultured thalamic neurons	activation	increased	Persico et al. (2006)
**5-HT**_**1A**_	mouse 8 DIV	cultured hippocampal neurons	activation	decreased	Rojas et al. (2017)
**5-HT**_**1A**_	mouse 7 DIV	cerebellar Purkinje cells	activation	increased	Kondoh et al. (2004)
**5-HT**_**1A**_	rat 14 DIV	cultured cortical neurons	activation	decreased	Sikich et al. (1990)
**5-HT**_**1A**_ **and 5-HT**_**7**_	mouse 8 DIV	cultured hippocampal neurons	activation	increased	Rojas et al. (2017)
**5-HT**_**2A**_ **receptors**	mouse 7 DIV	cerebellar Purkinje cells	activation	decreased	Kondoh et al. (2004)
**5-HT**_**3A**_	mouse P14-21	cortical pyramidal neurons	knockout	decreased	van der Velden et al. (2012)
**5-HT4R**	mouse E18	hippocampal neurons	activation	increased	Agrawal et al. (2019)
**5-HT6R**	mouse 2 DIV	striatal neurons	blocking	decreased	Pujol et al. (2020)
**Htr1a**	mouse P35	hippocampal pyramidal cells	blocking	increased	Ferreira et al. (2010)
**Htr1a**	mouse 7 DIV	serotonergic dorsal raphe neurons	activation	no change	Dudok et al. (2009)
**GABA receptors**					
**GABARs**	rat 9 DIV	neocortical neurons	blocking synthesis	decreased	Maric et al. (2001)
**GABARs**	mouse 2 weeks	cerebellar neurons	activation	increased	Baloyannis et al. (1983)
**GABARs**	mouse 7 DIV	cerebellar granule cells	activation	increased	Hansen et al. (1984)
**GABARs**	mouse 8 DIV	cerebellar granule cells	activation	increased	Borodinsky et al. (2003)
**GABARs**	mouse adult	adult-born hippocampal granule cells	activation	increased	Jagasia et al. (2009)
**GABA**_**A**_ **Rs**	mouse embryonic	hippocampal neurons	blocking	decreased	Barbin et al. (1993)
**GABA**_**A**_ **R γ2**	mouse adult	adult-born hippocampal granule cells	heterozygote mice	decreased	Ren et al. (2015)
**GABA**_**B**_ **Rs**	mouse P16	pyramidal neurons	knockout	decreased	Bony et al. (2013)
**GABA**_**B**_ **Rs**	rat E15	Neocortical neurons	blocking	decreased	López-Bendito et al. (2003)

The glutamate kainate receptors (KARs) have also been involved in dendritic growth. Overexpression of the GluK1 kainate subunit promoted inhibitory interneuron dendritic growth, while overexpression of the GluK2 kainate receptor promotes dendritic growth of excitatory pyramidal cells in the neocortex (Jack et al., 2019). The glutamate receptors NMDA type are also involved in dendritic development. *In vivo*, injection of NMDAR antagonists MK-801 or APV reduced dendritic growth of spinal cord motoneurons (Kalb, 1994). Furthermore, pharmacological antagonising of the GluN2B but not the GluN2A receptor reduced the basal dendrites of neocortical pyramidal cells (Gonda et al., 2020). On the other hand, a single-cell knockout of the glutamate receptor GluN2B in hippocampal granule cells and cortical spiny stellate cells did not alter the dendritic size of these cells (Espinosa et al., 2009). A reduction in dendritic growth has also been reported in glutamate receptor GluN2A knockout hippocampal neurons (Kannangara et al., 2014). Furthermore, the barrelette neurons in the principal trigeminal nucleus had an increased dendritic size in GluNR1 knockout mice (Lee et al., 2005). Blocking NMDARs with APV reduced dendritic length and branching of optic tectal neurons (Rajan and Cline, 1998) and spinal cord motoneurons (Inglis et al., 1998). Overexpression of the NMDARs subunit GluN3B increased dendritic complexity in spinal cord culture (Prithviraj and Inglis, 2008), and overexpression of the GluN2B mutant caused a decrease in dendritic length in neocortical neurons (Sceniak et al., 2019). These studies suggest a role for NMDARs in shaping dendritic trees.

How do glutamate receptors regulate dendritic growth? Generally, glutamate receptors regulate gene expression in neurons by activating various intracellular signalling cascades that phosphorylate transcription factors within the nucleus (Figure 2). On the other hand, it has been reported that glutamate can induce elongation of dendritic protrusions through group I mGluRs within minutes, suggesting that glutamate-induced dendritic growth is often way too rapid to be mediated by slow transcriptional events (Cruz-Martín et al., 2012). However, the exact mechanism by which glutamate induces rapid dendritic growth through group I mGluRs is unknown. One of the best-characterized signal cascades for glutamate-mediated growth regulatory process is the mitogen-activated protein kinase (MAPK) (Wang et al., 2007). MAPK is usually activated following sustained plasma membrane depolarisation, which triggers Ca^2+^ entry to the cell through glutamate receptors. Activity-dependent activation of the MAPK pathway in hippocampal neurons has been shown to elicit stable dendritic protrusion of new filopodia (Wu et al., 2001). Furthermore, the MAPK pathway is important for Ca^2+^ influx-mediated dendritic growth in neocortical neurons (Redmond et al., 2002). The Ca^2+^ permeable ionotropic glutamate receptor activates MAPKs through a signalling route involving the Ca^2+^-sensitive Ras-guanine nucleotide releasing factor, Ca^2+^/calmodulin-dependent protein kinase II (CaMKII), and phosphoinositide 3-kinase.

For the NMDARs, the activation of NMDARs triggers Ca^2+^ influx to neurons. Ca^2+^ influx to the cell activates Ca^2+^-dependent kinases to increase ERK phosphorylation (Figure 2). Among these kinases, the CaMKII was identified as a main Ca^2+^-sensitive kinase at the postsynaptic site. Protein kinase C (PKC) and phosphatidylinositol (phosphoinositide) 3-kinase (PI3-kinase) are also other candidate kinases that have been involved in NMDAR-mediated ERK phosphorylation. The high affinity of the calmodulin-binding sequence in PI3-kinase facilitates the association of calmodulin with PI3-kinase. Furthermore, ERK phosphorylation has been implemented in gene regulation. Interestingly, the phosphorylated form of ERK can regulate c-AMP response element binding protein (CREB). There is a strong connection between Ca^2+^ permeable glutamate receptor activation and CREB phosphorylation (Shaywitz and Greenberg, 1999; West et al., 2002). Activation of ERK through Ca^2+^ permeability of glutamate receptors has been shown to increase CREB phosphorylation (Mao et al., 2004). One of the targeted growth-promoting genes of CREB is the BDNF. Interestingly, in cortical neurons, Ca^2+^ influx triggers phosphorylation of CREB, which, by binding to a critical Ca^2+^ response element (CRE) within the BDNF gene, activates BDNF transcription (Tao et al., 1998). Mutation of BDNF CRE, an adjacent novel regulatory element, and a blockade of CREB function resulted in a dramatic loss of BDNF transcription (Tao et al., 1998). For the AMPARs, signalling for dendritic growth looks like the NMDARs. The AMPARs can induce MAPK/ERK phosphorylation (Wang et al., 2004). It has been shown that CaMKII and PI3-kinase are the two kinases that connect a pathway between AMPARs and ERK (Perkinton et al., 1999). Another mechanism by which AMPARs regulate dendrite growth is mediated through RasGRFs, which can form a complex with Ca^2+^-permeable AMPARs and subsequently guarantee a quick response of RasGRFs to Ca^2+^ signals derived from AMPARs to activate Ras-ERK (Tian and Feig, 2006). It is also important to mention that AMPARs have been coupled with p38 signaling, which could represent a determinant mechanism to decide whether nerve cells survive or die (Rivera-Cervantes et al., 2004). Kainate receptor-mediated Ca^2+^ influx has also been shown to trigger ERK phosphorylation in striatal neurons, and the active ERK1/2 cascade activates the downstream transcription factor CREB to participate in the regulation of gene expression (Mao et al., 2004). Taken together, these studies suggested that glutamate receptors ability to induce dendritic growth relies on Ca^2+^ permeability of the receptor and the subsequent increase in intracellular Ca^2+^, which triggers a dendritic growth-promoting signal cascade.

The trafficking of glutamate receptors is involved in dendritic growth. The discovery of the transmembrane AMPA receptor regulatory proteins (TARPs) paved the way to understand how AMPARs traffic to the plasma membrane and how the function of AMPARs is modulated (Straub and Tomita, 2012). By increasing trafficking and modulating the function of AMPARs, TARPS has also been implicated in dendritic growth (Hamad et al., 2014). In summary, overexpression of type I TARPs γ-2, γ-3, and γ-8, known to enhance AMPAR trafficking and insertion of AMPARs into the synaptic membrane, promoted dendritic growth (Figure 2). On the other hand, the overexpression of the TARP γ-4 subunit reduced dendritic growth (Hamad et al., 2014), possibly because it slows the channel opening and closing rates of AMPARs (Pierce and Niu, 2019). Type II TARPs γ-5 and γ-7 (also called non-TARPs), which potentiate the function of AMPARs but do not participate in the trafficking of AMPARs, did not affect the dendritic growth of neocortical neurons (Hamad et al., 2014). Interestingly, the TARP subunits, which increase the trafficking and insertion of new AMPAR subunits in the plasma membrane had elicited dendritic growth, whereas the TARP subunits, which only potentiate the existing receptors, did not affect dendritic growth. Furthermore, overexpression of the kainate receptor auxiliary subunit NETO1 but not NETO2 has been shown to promote dendritic growth of inhibitory interneurons (Jack et al., 2019). Furthermore, in neurons of the dorsal root ganglion, NETO2 plays a crucial and dynamic role in neurite outgrowth during early development and adulthood (Vernon and Swanson, 2017). Thus, these studies suggest that enhancing the AMPARs or kainate receptor density on the plasma membrane could also be another mechanism by which neurons regulate their dendritic growth.

Metabotropic glutamate receptors are also involved in dendrite growth (Figure 2). For instance, in group I metabotropic glutamate receptor 5 (mGluR5) knockout mice, dendritic growth of neocortical spiny stellate neurons was increased (Ballester-Rosado et al., 2010). On the other side, the activation of the metabotropic glutamate receptor mGluR1 decreased cerebellar Purkinje cells dendritic growth, and it does not require activation of phospholipase C or protein kinase C (Sirzen-Zelenskaya et al., 2006). It has been suggested that mGluR1 and mGluR5 receptor coupling to ERK occurred via mechanisms independent of PI3K activity and intracellular and/or extracellular Ca^2+^ concentration (Thandi et al., 2002). Therefore, it has been shown that these two closely related mGluR receptors utilize different G proteins to cause ERK activation and may recruit different tyrosine kinases to facilitate this response (Thandi et al., 2002).

#### 2.1.2 GABA

**FIGURE 3:**
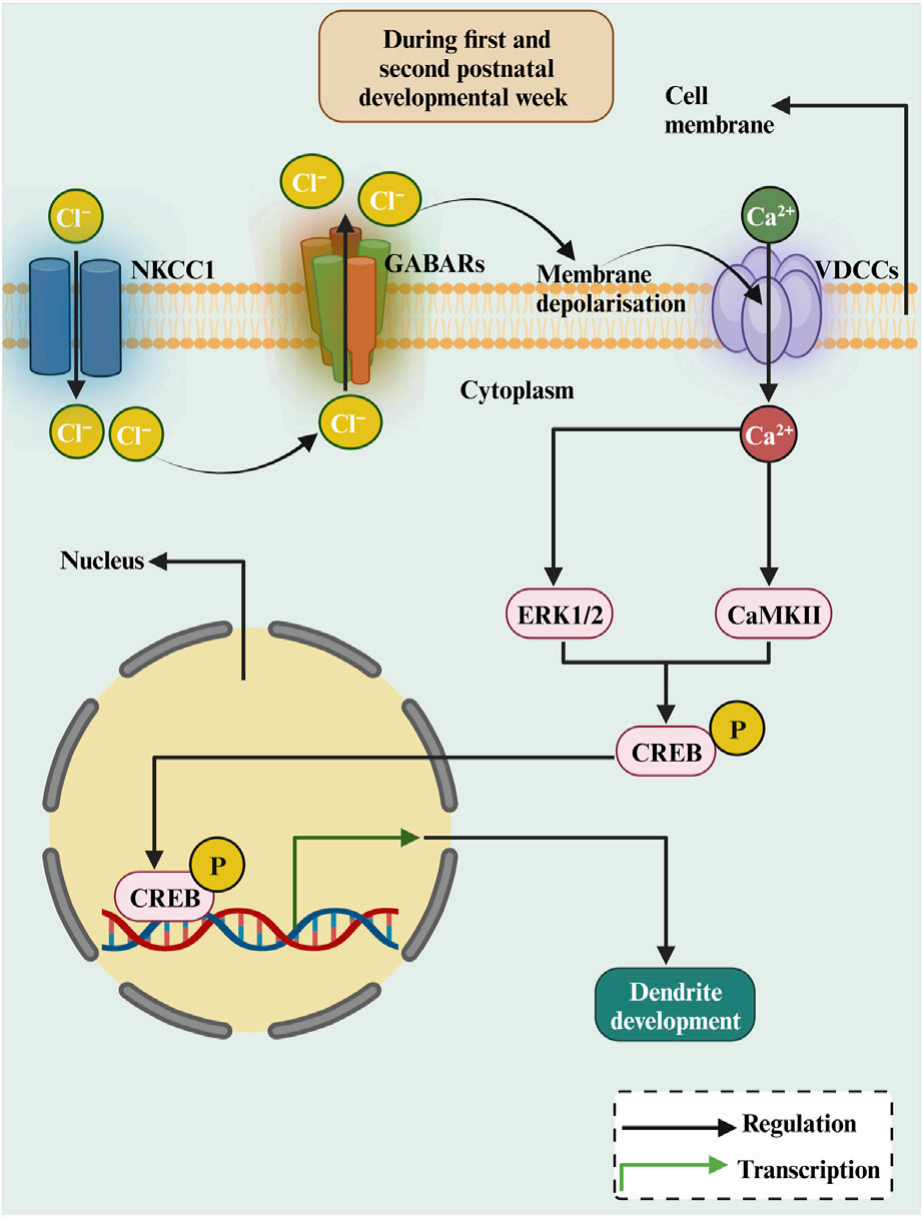
The diagrammatic representation of the roles of depolarizing GABA in dendritic growth during early development. In the immature brain, the intracellular Cl− concentration is relatively high, as Cl− transport over the membrane is dominated by NKCC1. Activation of GABA_A_Rs results in an outflow of Cl− resulting in membrane depolarization. This results in Ca^2+^ inflow and activating CREB by ERK1/2 or CaMKII, stimulating transcription of the BDNF gene, which is crucial for dendritic growth and development.

The GABA excitation/inhibition shift occurs during brain development between the first and second postnatal developmental week (Ben-Ari et al., 2007; Kirmse et al., 2015; Luhmann, 2021). The depolarizing effect of GABA, which occurs only in early development in developing neurons, is due to the expression of the cotransporter NKCC1, which causes an efflux of Cl^−^ ions following the activation of GABA_A_Rs (Blaesse et al., 2009). Thus, the activation of GABA_A_Rs in early development depolarises the plasma membrane, leading to Ca^2+^ influx through the voltage-dependent Ca^2+^ channels (VDCCs) (Leinekugel et al., 1997). This will raise intracellular Ca^2+^ levels (Tozuka et al., 2005) and thereby promote dendritic growth (Figure 3). Studies investigating the role of GABA and its receptors in dendritic growth are summarised in Table 1. GABA treatment of rat cortical neurons increased dendritic length and branching (Maric et al., 2001). Furthermore, blocking GABA_A_Rs with bicuculline decreased dendrite length and branching of hippocampal neurons (Barbin et al., 1993). In cerebellar culture neurons, feeding medium enriched with GABA enhanced dendritic growth and dendritic arborization (Baloyannis et al., 1983). Furthermore, it has been shown that GABA serves as a trophic factor for cerebellar granule cells (CGCs) (Hansen et al., 1984). The effect of GABA on CGCc dendrite growth was induced by Ca^2+^ influx through the activation of L-type VDCCs, as it was prevented by the blocker nifedipine (Borodinsky et al., 2003). Interestingly, GABA-induced dendritic growth of CGCs was mediated by GABA_A_Rs activation and requires Ca^2+^ influx and subsequent activation of CaMKII and ERK1/2 pathways (Borodinsky et al., 2003). Moreover, the adult hippocampal newly born granule cells dendrites were smaller in the γ2 GABA_A_Rs heterozygote mice (Ren et al., 2015), as GABA_A_R-mediates depolarisation of neurons and activate a Ca^2+^-dependent signalling cascade that promotes the expression of the BDNF gene and BDNF release (Baldelli et al., 2002). Furthermore, depolarising GABA is involved in the phosphorylation of CREB during the second postnatal week of newly generated neurons in the adult hippocampus, and that results in an enhancement of dendritic growth (Jagasia et al., 2009). This occurs because CREB signalling converges onto the BDNF pathway for promoting dendritic growth (Tao et al., 1998). Together, these studies suggest that GABA_A_R activation induces Ca^2+^ influx, which triggers the activation of the CaMKII and ERK1/2 pathways and this results in CREB phosphorylation and the subsequent transcription of BDNF to promote dendritic growth (Figure 3). However, this effect is restricted only to early postnatal development when GABA is still excitatory. At the end of the postnatal week, the dendritic growth-prompting action of GABA will reach an end as GABA becomes hyperpolarizing (inhibitory), and that inhibitory action is indispensable for the control of network activity.

The role of GABA_B_Rs in dendritic growth is still not well-documented. However, some studies have reported an effect of the GABA_B_Rs on dendrite growth. For example, blocking the GABA_B_R in organotypic cultures reduced the dendritic growth of migratory inhibitory GABAergic neurons (López-Bendito et al., 2003). The removal of GABA_B_R *in utero* resulted in smaller dendrites of neocortical pyramidal cells due to disruption of cAMP/LKB1 signalling (Bony et al., 2013). These studies suggest that GABA_B_Rs are also involved in dendritic growth in early development because, during the GABA_B_Rs activation, they lack coupling to potassium channels and thus exert a non-hyperpolarizing action.

#### 2.1.3 Serotonin

**FIGURE 4:**
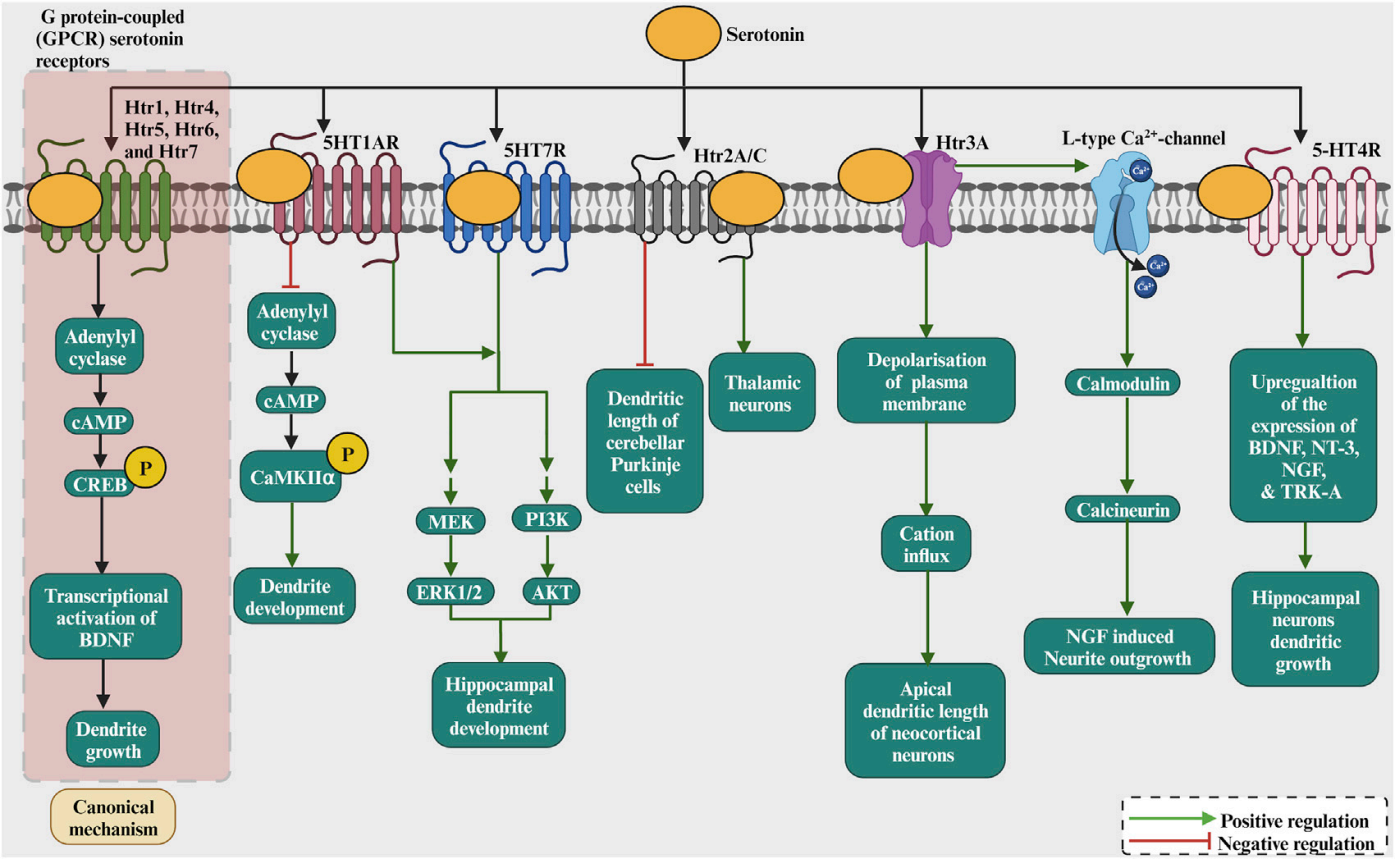
The role of serotonin as a neurotransmitter by binding to receptors such as 5HT1R, Htr2A/C, Htr3A, 5HT4R, which modulates adenylate cyclase and phosphorylating CREB/CaMKII. This results in the transcription of BDNF/NT3/NGF/Trk-A and activation of CREB by ERK1/2 or others that regulate dendritic growth and development. Serotonin induces the increase in intracellular Ca^2+^ via activation of 5-HT3A receptors and voltage-gated Ca^2+^ channels and subsequent activation of calmodulin and calcineurin which enhances NGF-induced neurite outgrowth. The 5-HT4R stimulation increased hippocampal neurons dendritic growth as it upregulates the expression of the neurotrophic factors BDNF, NT-3, NGF and also the TRK-A receptor.

The neurotransmitter serotonin has also been involved in the dendritic growth process (Figure 4; Table 1). Serotonin application to rat cortical neurons has been shown to inhibit dendritic growth and branching (Sikich et al., 1990). On the other hand, serotonin has been shown to increase dendritic growth and branching of cultured thalamic neurons (Persico et al., 2006). Moreover, in organotypic cultures, cerebellar Purkinje cells dendritic growth was promoted by serotonin through 5-HT1A receptors but inhibited by serotonin through 5-HT2A receptors (Kondoh et al., 2004). However, the inhibition of serotonin synthesis, using DL-P-chlorophenylalanine (PCPA), a reversible inhibitor of 5-HT synthesis, during embryonic developmental stage, reduced dendritic growth of adult cortical pyramidal neurons (Vitalis et al., 2007). Serotonergic receptors mediate the dendritic growth process. Activating the serotonin receptor Htr1A with 8-OH-DPAT increased dendritic length and branching in cultured embryonic thalamic neurons in mice (Persico et al., 2006). Later, activation of Htr1A by 8-OH-DPAT for 7 days did not affect dendritic growth in mouse dorsal raphe nuclei slice cultures (Dudok et al., 2009). Similarly, no morphological changes have been revealed in Htr1A knockout mice (Heisler et al., 1998). In addition, the pharmacological blockade of serotonin receptor 1A (Htr1a) increased hippocampal pyramidal dendritic growth at P35 (Ferreira et al., 2010). The Htr1a activation reduces the cAMP levels by inhibiting adenylyl cyclase and increasing autophosphorylation of CaMKIIα, which restricts dendritic growth. The Htr1B receptor seems not to be involved in dendritic growth since Htr1B knockout mice presented with no morphological abnormality (Saudou et al., 1994). Furthermore, activating the 5-HT1a and 5-HT7 receptors increased the dendritic growth of cultured hippocampal neurons (Rojas et al., 2017). The activation of 5-HT1AR and 5-HT7R promotes the growth of short secondary dendrites and triggers ERK1/2 and AKT phosphorylation through MEK and PI3K activation, respectively. Studies on the Htr2A/C receptor have shown different effects on neurite growth. For example, activation of the Htr2A/C receptor decreases the total dendritic length of cerebellar Purkinje cells (Kondoh et al., 2004). However, activation of the Htr2A/C enhanced dendritic growth of cultured embryonic mouse thalamic neurons in mice (Persico et al., 2006). Among the serotonin receptors, Htr3 is the only ionotropic receptor subtype. The activation of the Htr3 receptor with m-CPBG enhanced the dendritic growth of cultured rat cortical neurons (Vitalis and Parnavelas, 2003). No abnormalities were found in Htr3A knockout mice (Walstab et al., 2010), however, an enhancement of apical dendrite length has been reported in the neocortical neurons of Htr3A knockouts (van der Velden et al., 2012). Therefore, how do serotonin receptors modulate dendritic growth? First of all, as the studies above suggested, serotonin has a different growth action for dendritic growth, and that effect depends on the nature of the receptor (Figure 4). For instance, the Htr1 and Htr5 receptor families are inhibitory. On the other hand, the Htr2, Htr3, Htr4, Htr6, and Htr7 receptor families are excitatory. Furthermore, the stimulation of 5-HT4R with the agonist RS67333 had increased hippocampal neurons dendritic growth (Agrawal et al., 2019) as it upregulates the expression of the neurotrophic factors BDNF, NT-3, NGF as also the TRK-A receptor. Moreover, Htr3 is ionotropic and could depolarize the plasma membrane, resulting in cations influx. The other serotonin receptors are G protein-coupled (GPCR). For example, Htr1, Htr4, Htr5, Htr6, and Htr7 modulate cAMP activity, and by increasing cAMP activity, CREB can be phosphorylated, targeting BDNF transcription. For the Htr3 receptor, serotonin-induced increases in intracellular Ca^2+^, which are mediated by 5-HT (3) receptors and L-type Ca^2+^ channels, and subsequent activation of calmodulin and calcineurin have been shown to enhance NGF-induced neurite outgrowth (Homma et al., 2006). The serotonin receptors activating the G protein have a dendritic growth signalling pathway (Figure 4). For instance, the serotonin receptor Htr1A activation leads to a number of neurotrophic events by activating a series of signal transduction molecules and these effect s has been shown to be mediated through G protein (Fricker et al., 2005). Finally, serotonin has been shown to induce the expression of growth factors, in particular BDNF (Vaidya et al., 1997). Taken together, these studies suggest that serotonin modulate dendritic growth. The action of serotonin depends on the nature of the activated receptor as well as the activated signaling cascade.

### 2.2 Neurotrophins

Neurotrophins are a family of closely related proteins initially identified as survival factors for sensory and sympathetic neurons and have since been shown to regulate many aspects of neuronal survival, development, and function in the peripheral and the central nervous systems. Neurotrophins consist of nerve growth factor (NGF), brain-derived growth factor (BDNF), neurotrophin 3 (NT-3), and neurotrophin 4 (NT-4). Individual neurotrophins have been shown to activate the p75 neurotrophin receptor (p75NTR), a member of the tumour necrosis factor receptor superfamily (McAllister et al., 1999). In addition, neurotrophins have been shown to activate one or more of the three known members of the tropomyosin-related kinase (Trk) family of receptor tyrosine kinases (TrkA, TrkB and TrkC). Neurotrophins have been shown to play a key role in regulating dendritic architecture in neurons. Studies investigating the role of neurotrophins in dendritic growth are summarised in Table 2. BDNF is perhaps the most extensively studied molecule known to influence cortical neurons dendritic development and its receptor, TrkB, is broadly expressed in the developing and adult mammalian brain. Early studies carried out in organotypic cultures showed the contribution of BDNF to dendritic development, where is has been shown to increase the dendrite complexity of cortical pyramidal neurons by increasing total dendritic length, the number of dendritic segments, and the number of primary dendrites (McAllister et al., 1995; Niblock et al., 2000). Interestingly, BDNF exerted a growth effect after 1 day of treatment. Furthermore, the effect of BDNF on dendritic growth was specific as it was pronounced in basal but not apical dendrites. The overexpression of BDNF in neocortical pyramidal neurons elicited dramatic sprouting of basal dendrites, accompanied by a regression of dendritic spines (Horch et al., 1999). In early development, the overexpression of BDNF or NT4 in neocortical pyramidal cells increased dendritic length and number of segments without affecting maximum branch order and number of primary dendrites, suggesting that early in development BDNF probably accelerates dendritogenesis in an autocrine fashion (Wirth et al., 2003). NT-4 and NT-3 could, in part, mediate dendritic growth and branching of cortical pyramidal neurons in earlier developmental stages as it is expressed at an early age during cortical development and also signals through the TrkB receptor (Timmusk et al., 1993). In addition, in the visual cortex, BDNF released from dendrites and cell bodies acted directly on nearby recipient neurons to increase dendritic branching in a distance-dependent manner (Horch and Katz, 2002), suggesting that BDNF can control dendritic structure in a spatially restricted manner. Furthermore, using a conditional mouse mutant essentially lacking BDNF, it has been shown that BDNF remarkably enhanced dendritic growth of cultured striatal neurons but not of those of hippocampal neurons, suggesting that the differential responsiveness to BDNF is part of a neurone-intrinsic program (Rauskolb et al., 2010). Furthermore, the overexpression of BDNF in the dendritic structure of granule cells of the hippocampal dentate gyrus increases their dendritic complexity (Tolwani et al., 2002). BDNF has also been shown to modulate the dendritic architecture in cortex and hippocampus in a cell type-specific manner, suggesting that diverse levels of responsiveness to BDNF among different populations of hippocampal and cortical neurons within the same brain area (Zagrebelsky et al., 2018). Taken together, these studies suggest that neurotrophins exert their growth-promoting effect in a cell-type-specific and spatially restricted manner.

**TABLE 2:** Summary of the studies conducted on the role of neurotrophins and their receptors in dendritic growth. Days *in vitro* (DIV). Postnatal day (P).

Protein	Animal and age	System	Strategy	Growth effect	Reference
**BDNF**	ferret P25–28	cortical pyramidal cells	overexpression	increased	Horch et al. (1999)
**BDNF**	rat 10 DIV	cortical pyramidal cells	overexpression	increased	Wirth et al. (2003)
**BDNF**	mouse 8 weeks	hippocampal neurons	overexpression	increased	Tolwani et al. (2002)
**BDNF**	mouse 2 months	cultured striatal neurons	knockout	decreased	Rauskolb et al. (2010)
**BDNF**	mouse 8 weeks	hippocampal neurons	knockout	decreased	Zagrebelsky et al. (2018)
**BDNF**	ferret P25–28	cortical pyramidal cells	co-culture	increased	Horch and Katz (2002)
**BDNF**	rat 7 DIV	neocortical neurons	stimulation	increased	Dijkhuizen and Ghosh (2005)
**BDNF**	rat 7 DIV	hippocampal neurons	stimulation	increased	Kwon et al. (2011)
**BDNF**	rat 7 DIV	hippocampal neurons	stimulation	increased	Chandía-Cristi et al. (2023)
**BDNF**	rat 9 DIV	hippocampal neurons	stimulation	increased	González-Gutiérrez et al. (2020)
**BDNF**	rat DIV2	cortical neuron	stimulation	increased	Miyamoto et al. (2006)
**p75NTR**	mouse 20 DIV	hippocampal pyramidal cells	knockdown	increased	Zagrebelsky et al. (2005)
**p75NTR**	mouse 3 weeks	cultured sympathetic neurons	overexpression	increased	Courter et al. (2016)
**TrkB**	ferret P14	cortical neurons	overexpression	increased	Yacoubian and Lo (2000)
**TrkB**	ferret P14	cortical pyramidal cells	activation	increased	McAllister et al. (1995)
**TrkB**	rat P10	cortical pyramidal cells	activation	increased	Niblock et al. (2000)
**TrkB**	mouse 6 DIV	Dissociated hippocampal neurons	activation	increased	Cheung et al. (2007)
**TrKB**	mouse P0	neocortical neurons	knockout	decreased	Gates et al. (2000)
**TrkB**	mouse P42	neocortical neurons	Knockout	decreased	Xu et al. (2000)
**TrkC**	mouse P14, 21, 90	cerebellar Purkine cell	knockout	decreased	Joo et al. (2014)
**TrkC**	mouse P14, 21 and 90	cerebellar Purkinje cells	Knockout	decreased	Joo et al. (2014)

Interestingly, altering TrkB expression has also been shown to influence dendrite growth. For instance, conditional deletion of TrkB in cortical pyramidal neurons induced dendritic retraction and neuronal loss, suggesting that BDNF/TrkB signalling is required for the development of neocortical neurons and maintenance of certain neuronal subtypes in the adult neocortex (Gates et al., 2000). Furthermore, in the ferret visual cortical slices, the overexpression of full-length and truncated TrkB receptors exerted differential effects on dendritic branching, with the full-length TrkB increasing proximal dendritic branching, whereas truncated TrkB promoted net elongation of distal dendrites (Yacoubian and Lo, 2000). Thus, the morphological effects of each receptor isoform were complementary to one another. A study using TrkB knockout mice indicated that TrkB signaling is important for the regulation of dendritic branching of cortical pyramidal neurons *in vivo* (Xu et al., 2000). The effects of p75NTR on dendrite growth depend on the cell type investigated with its overexpression in mouse-cultured sympathetic neurons increasing dendritic length and branching (Courter et al., 2016). In contrast, in hippocampal pyramidal cells, dendritic growth was increased in p75NTR knockout organotypic cultures (Zagrebelsky et al., 2005). It has been shown that p75NTR constitutively activated RhoA and negatively modulated the length of neurites and filopodia (Gehler et al., 2004). Furthermore, in TrkC knockout mice, the dendritic complexity of cerebellar Purkinje cells was reduced, suggesting that NT-3/TrkC levels are critical for their signaling (Joo et al., 2014). These data suggest that neurotrophin and its receptors play a pivotal role in dendritic growth, and their effect depends on the cell type.

**FIGURE 5:**
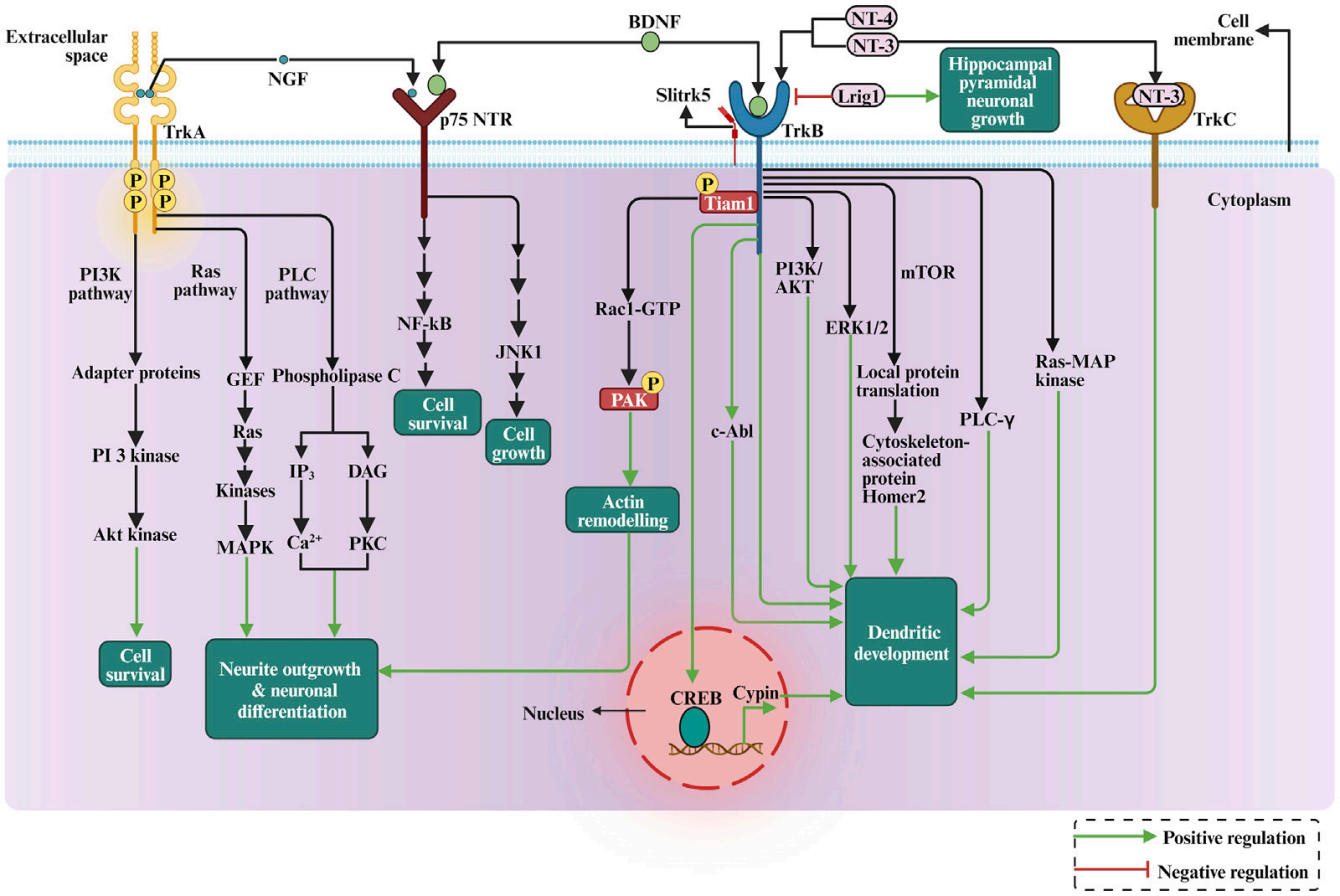
The depiction of the role of neurotrophins in dendritic growth and development involves the cellular signalling pathways, PI3K pathway, RAS-MAP kinase pathway, mTOR signalling, and actin cytoskeleton rearrangement via Rho GTPases.

Hence, how do neurotrophins binding to their receptors affect dendritic growth? Trk receptors cytoplasmic domains contain several tyrosine phosphorylation sites that recruit intermediates in intracellular signalling cascades (Figure 5). For instance, neurotrophin signalling has been shown to regulate actin filament dynamics by modulation of Rho GTPase family proteins and other actin remodelling proteins (Miyamoto et al., 2006; Cheung et al., 2007; Shirazi Fard et al., 2010; Hedrick et al., 2016). In addition, BDNF leads to the rapid activation of PI3-kinase, MAP kinase, and PLC-gamma in cortical neurons, and pharmacological inhibition of PI3-kinase and MAP kinase in dissociated cell cultures and cortical slice cultures suppresses the ability of BDNF to induce dendrite formation, suggesting that BDNF induces primary dendrite formation via activation of the PI3-kinase and MAP kinase pathways (Dijkhuizen and Ghosh, 2005). Applying BDNF to dissociated hippocampal neurons has increased neuronal cypin expression. The cypin promoter region contains putative conserved cAMP response element (CRE) regions, which have been found to be recognized and activated by the CRE-binding protein (CREB). It has been shown that BDNF promoted increases in proximal dendrites via CREB-dependent transcriptional regulation of cypin (Kwon et al., 2011). A more specific mechanism in which TrkB receptor signalling regulates dendrite growth has recently been suggested, with the non-receptor tyrosine kinase c-Abl as a possible candidate regulator of this process, as it has been implicated in tyrosine kinase receptors signalling and trafficking and consequently the regulation of dendritogenesis (Figure 5). BDNF/TrkB-dependent activation of c-Abl has been shown to be a novel and essential mechanism in TrkB signalling, which is required for branching and growth of dendrites (Chandía-Cristi et al., 2023). Furthermore, BDNF-promoted c-Abl activation did not require the signaling pathways ERK1/2, phosphoinositide 3-kinase (PI3K) and PLC-γ signalling pathways, suggesting that c-Abl acts through an independent pathway downstream of TrkB (Chanda-Cristi et al., 2023). Furthermore, following TrkB activation, the receptor undergoes endocytosis and transport to the neuronal soma. Surprisingly, in hippocampal neurons, the internalised neurotrophin bound to its Trk receptor continues to signal in endosomes and trigger the activation of Ras-MAP kinase, PLC-γ, and PI3K pathways (González-Gutiérrez et al., 2020). Therefore, the endosomal pathway is required for the signaling cascade initiated by BDNF and its receptors on the plasma membrane to modulate BDNF-dependent genes and neuronal dendritic growth mediated by the CREB transcription factor (González-Gutiérrez et al., 2020). Consequently, the TrkB receptor can be recycled into the plasma membrane or degraded by lysosomes (Gonzalez et al., 2016). It has been proposed that Rab11-dependent dendritic recycling provides a mechanism to retain TrkB in dendrites and to increase local signalling to regulate arborization (Lazo et al., 2013). Recent work has shown that the recycled TrkB can be required for long-distance induction of dendritic growth mediated through dynein-dependent BDNF-TrkB-containing endosomal transport mechanism (Moya-Alvarado et al., 2023). Furthermore, BDNF-induced dendritic growth leads to activation of the PI3K/AKT and ERK1/2 signaling pathways, as well as activation of local protein translation by mTOR (mammalian target of rapamycin) kinase (Schratt et al., 2004; Jaworski et al., 2005; Kumar et al., 2005; see also Figure 5). The local translation of the Homer2 cytoskeleton-associated protein has been shown to have important implications for growth cone dynamics and dendritic development (Schratt et al., 2004). Other studies demonstrated Trk receptors engagement with specific Leucine-rich repeat (LRR) transmembrane proteins to modulate dendritic growth. For instance, the LRR protein Lrig1 was found to interact physically with TrkB and attenuate BDNF signalling, thereby promoting hippocampal pyramidal neuronal growth (Alsina et al., 2016). In addition, Slitrk5 mediates BDNF-dependent TrkB receptor trafficking and signalling, suggesting that Slitrk5 acts as a TrkB co-receptor (Song et al., 2015, see also Figure 5). Thus, LRR proteins interaction with Trk signalling delivered further mechanisms by which neurotrophins regulate dendrites development.

The neurotrophin function appears to be linked to TRPC signaling in several ways. The TRPC is a family of transient receptor potential cation channels in animals and they regulate brain development, including neural progenitor proliferation, neurogenesis, neuron survival, axon guidance, and dendritic morphology (Tai and Jia, 2017). Some TRPC family members have been shown to influence dendritic growth (Tai et al., 2008; Puram et al., 2011; Jeon et al., 2013). Interestingly, the TRPC3 channels are necessary for BDNF to increase dendritic spine density. The activation of TrkB through BDNF leads to a TRPC3-dependent cation influx in CNS neurons *in vivo* (Li et al., 1999). Thus, TRPC channels emerge as novel mediators of BDNF-mediated dendritic remodeling through the activation of a slowly developing and sustained membrane depolarization (Amaral and Pozzo-Miller, 2007). Furthermore, the Ca^2+^ influx through TRPC5 induced by NT-3 inhibited neuronal dendritic growth by activating CaMKIIα. In contrast, the Ca^2+^ influx through TRPC6 induced by NT-4 promoted the dendritic growth (He et al., 2012). Thus, TRPC5 acts as a novel and specific mediator for NT-3 to regulate dendrite development through CaMKIIα. Besides regulating the sensory function of the nervous system, some TRPC family members are necessary for neurotrophins to promote dendritic growth.

### 2.3 Extracellular matrix proteins

**FIGURE 6:**
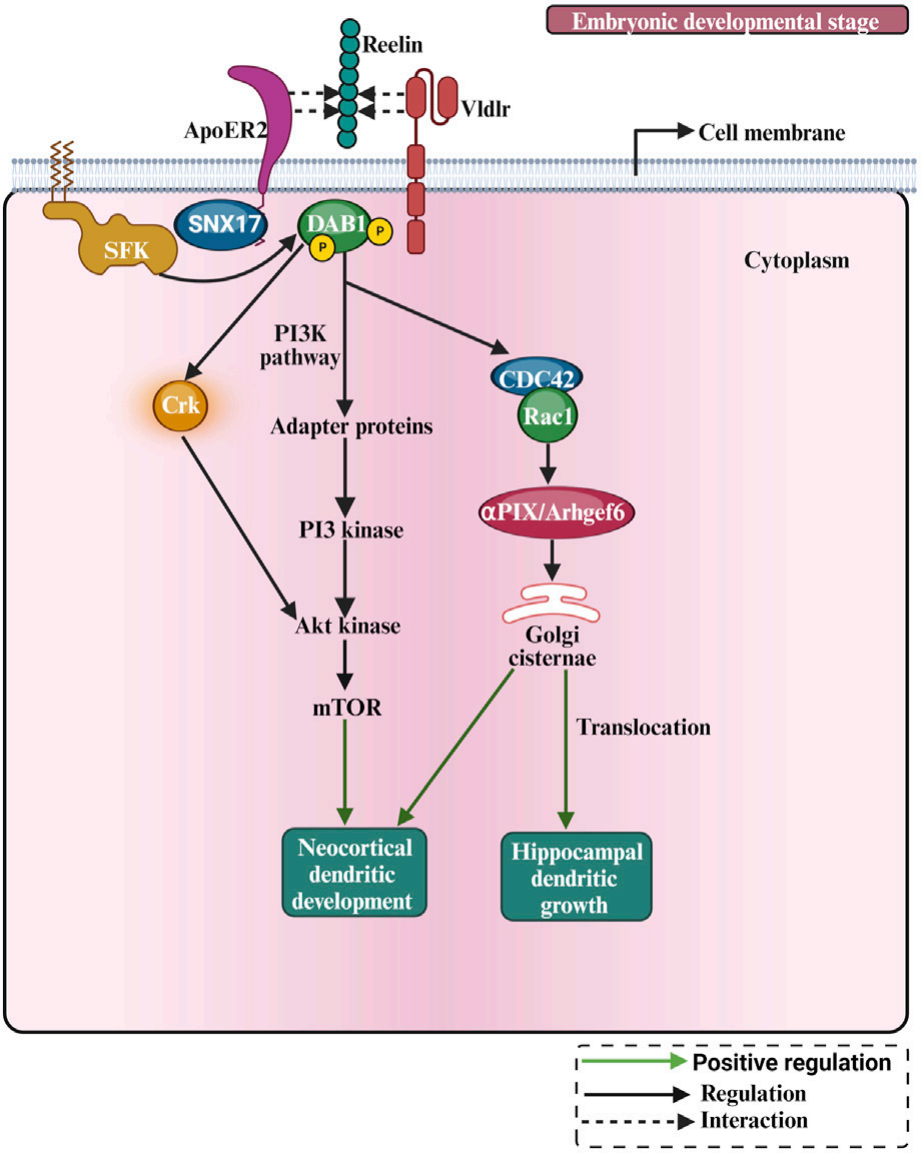
The pictorial representation of reelin involved in dendritic growth and development. Reelin is involved in cellular pathways such as the PI3K pathway. During early postnatal development, the Crk adaptor proteins and the PI3K-Akt-mTOR pathway contribute to Reelin activity by promoting protein translation, and dendrite outgrowth.

In early development, the ECM regulates neuronal morphology, cell shape, differentiation, migration, and morphogenesis of neural tissues (Long and Huttner, 2019). Most well-known ECM proteins influence neurite outgrowth and axon pathfinding by integrin-ECM interaction mechanisms (Myers et al., 2011). Studies investigating the role of the ECM in dendritic growth are summarised in Table 3. Laminin was found to promote neurite outgrowth in cultured neurons (Lander et al., 1985), and it regulates neurite growth and neuronal migration via integrin signalling through the AKT/GSK-3beta pathway (Chen et al., 2009b). Moreover, the activation of integrins has also been shown to regulate dendrite growth in retinal neurons (Ivins et al., 2000). Furthermore, a selective loss of integrin β1 in excitatory hippocampal neurons reduces dendritic size and complexity (Warren et al., 2012). Moreover, the integrin α3 stabilizes the dendritic tree of hippocampal neurons through an Arg nonreceptor tyrosine kinase, which activates p190RhoGAP, and that, in turn, inhibits the RhoA GTPase and thereby stabilizes the dendritic tree (Kerrisk et al., 2013). The classical example of ECM regulating neuronal growth is the role of reelin in neocortical development (Figure 6). Reelin is a well-known ECM protein that controls several aspects of mammalian brain development and function. It controls neuronal migration and layer formation in the developing cerebral cortex (Caviness, 1976). Reelin signaling, through its membrane receptors (Bock and May, 2016), guides the migration of newborn neurons and orchestrates the development of cortical layers. The canonical Reelin signaling cascade requires direct binding of reelin to ApoER2 and VLDLR and activation of the intracellular adapter protein disabled-1 (DAB1) by tyrosine phosphorylation (D’Arcangelo et al., 1999; Trommsdorff et al., 1999). The most important function of reelin in early development is to guide the migration of newborn neurons and to control proper cortical layer development (for a review, see (Förster et al., 2010; Jossin, 2020)). Reelin exerts an opposing effect on dendritic growth depending on the developmental stage. During the embryonic developmental stage (Figure 6), reelin promotes dendritic growth and branching of embryonic pyramidal cells in dissociated hippocampal cell cultures of reeler mutant mice (Niu et al., 2004), and it increases dendritic growth of hippocampal neurons (Jossin and Goffinet, 2007; Matsuki et al., 2008). Moreover, neocortical pyramidal cell apical dendrites failed to extend and contact the marginal zone when reelin signalling was suppressed (Olson et al., 2006) and reorganized their dendrites in a tangential orientation to the marginal zone (O’Dell et al., 2015). Furthermore, reelin-induced branching of the leading processes of migrating neurons and the apical processes of radial glial cells (Chai et al., 2015). Furthermore, deletion of the reelin’s C-terminal region around E14.5 reduced the apical dendritic length of pyramidal cells around P7 (Kohno et al., 2015). Finally, reelin was found to specify the molecular identity of the pyramidal neuron distal dendritic compartment through enrichment of HCN1 and in the distal tuft dendrites of hippocampal CA1 neurons (Kupferman et al., 2014), however, conditional reelin knockout mice did not show an altered HCN1 distribution in CA1 neurons (Meseke et al., 2018). In turn, during postnatal development, an opposite effect of reelin on dendritic growth was reported. The N-terminal region of reelin restricted dendritic growth of neocortical apical pyramidal neurons (Chameau et al., 2009), and morphometric analysis of interneurons in the adult reeler neocortex and hippocampus revealed hypertrophic growth with longer dendritic branches in reeler when compared with wild-type interneurons (Yabut et al., 2007). Furthermore, newly generated neurons in the adult hippocampus respond to reelin overexpression with faster development of the dendritic tree within the first 2 weeks, while the complexity of the dendritic was reduced after 8 weeks (Teixeira et al., 2012). Finally, knocking out reelin conditionally in early postnatal development increased inhibitory interneuron dendritic growth (Hamad et al., 2021b) due to the absence of GABA_B_R signalling (Hamad et al., 2021a). These data suggest that reelin has opposing effects on morphological neuronal maturation during embryonic when compared with postnatal dendritic development. Differential effects of the different proteolytic fragments of reelin explain the discrepancy between the roles of reelin during embryonic *versus* postnatal development. During early embryonal development, reelin is secreted by Cajal-Retzius (CR) cells, which disappear around P14 by undergoing selective cell death through apoptosis, and it remains exclusively expressed by inhibitory interneurons (Pohlkamp et al., 2014). There is an important biochemical basis for the function of reelin proteolysis in brain development and function (Kohno et al., 2009). After secretion from CR cells, reelin cleavage produces at least five fragments that could be detected using antibodies against N-terminal, central, and C-terminal reelin epitopes (Jossin et al., 2004). The secreted reelin fragments containing the C-terminal fragment appeared to remain localized within the marginal zone (Jossin et al., 2007). Because the C-terminus of reelin is necessary for promoting dendritic growth (Kohno et al., 2015), pyramidal cell apical dendrites, localized in the vicinity of the marginal zone, benefit from locally deposited C-terminal fragments to promote their dendritic growth. It has been shown that the cleaved N-terminal fragment is transported to distant regions of the superficial layers (Koie et al., 2014), and acts oppositely to the C-terminal fragment, i.e., as a growth-limiting signal for dendritic growth (Chameau et al., 2009). Thus, the observed discrepancies of the reelin effects might be due to the differential action of the various reelin proteolytic fragments. Hence, which signal mechanism does reelin activate to promote dendritic growth? The main component of the reelin signalling pathway which were documented for dendritic growth are the VLDLR, ApoER2, Dab1 and SFK (Niu et al., 2004; Olson et al., 2006; Jossin and Goffinet, 2007; see also Figure 6). In cultured hippocampal neurons, it has been shown that Crk family proteins are important downstream components of the reelin signalling pathway in regulating postnatal hippocampal dendritic growth (Matsuki et al., 2008). Furthermore, it has been shown that reelin signalling requires PI3-kinase, Akt and mTor for proper neocortical dendritic development (Jossin and Goffinet, 2007; see also Figure 6). Furthermore, reelin regulates the extension of the Golgi along the dendritic shaft in the cortex and hippocampus (Matsuki et al., 2010; O’Dell et al., 2012). The Cdc42/Rac1 guanine nucleotide exchange factor αPIX/Arhgef6 promoted Golgi cisternae into developing dendrites of hippocampal neurons, and reelin treatment further increased the αPIX-dependent effect, suggesting that αPIX may promote dendritic Golgi translocation, as a downstream component of a reelin-modulated signaling pathway (Meseke et al., 2013). Furthermore, there is a connection between endocytic trafficking and reelin receptor signalling. When the SNX17 (an ApoER2 binding partner of sorting) interacts with the ApoER2 cytoplasmic domain, it facilitates receptor exit from a Rab5 to a Rab11 endosomal compartment and its recycling to the cell surface (Sotelo et al., 2014). This mechanism influences ApoER2 cell surface levels and reelin-induced signalling for dendritic growth (Sotelo et al., 2014). Recently, it has been shown that there is an interaction between reelin and neuropilin-1 (Nrp1) which is required for normal dendritic development in superficial-layer neurons (Kohno et al., 2020). Taken together, these studies suggest that reelin regulates dendritic growth by using different signalling pathways to guarantee proper neuronal maturation.

**TABLE 3:** Summary of the studies on the role of ECM proteins and their receptors in dendritic growth. Days *in vitro* (DIV). Postnatal day (P).

Protein	Animal and age	System	Strategy	Growth effect	Reference
**EphA7**	mouse P10	cortical pyramidal cells	knockdown	increased	Clifford et al. (2014)
**Integrin**	rat E18	retinal neurons	activation	increased	Ivins et al. (2000)
**Integrin β1**	mouse 15 DIV	hippocampal neurons	knockout	decreased	Warren et al. (2012)
**Integrin α3**	mouse P42	hippocampal neurons	knockout	decreased	Kerrisk et al. (2013)
**Laminin**	rat	superior cervical ganglion cells	activation	increased	Lander et al. (1985)
**Laminin γ1**	mouse E18	cortical neurons	knockout	decreased	Chen et al. (2009b)
**Reelin**	mouse 10 DIV	cortical interneurons	knockout	increased	Hamad et al. (2021b)
**VLDLR and ApoER2**	mouse 5 DIV	dissociated hippocampal neurons	activation	increased	Matsuki et al. (2008)
**VLDLR and ApoER2**	mouse E18	Dissociated cortical neurons	activation	increased	Chai et al. (2015)
**VLDLR and ApoER2**	mouse P70	hippocampal granule cells	activation	increased	Teixeira et al. (2012)
**VLDLR and ApoER2**	mouse P14	hippocampal pyramidal cells	activation	decreased	Niu et al. (2004)
**VLDLR and ApoER2**	mouse E18	hippocampal neurons	activation	increased	Jossin and Goffinet (2007)
**VLDLR and ApoER2**	mouse P7	cortical slice	blocking	decreased	Kohno et al. (2015)
**VLDLR and ApoER2**	mouse 7 DIV	cortical organotypic cultures	blocking	increased	Chameau et al. (2009)
**VLDLR and ApoER2**	mouse P14	cortical brain slices	blocking	increased	Yabut et al. (2007)
**VLDLR and ApoER2**	mouse P70	hippocampal granule cells	blocking	increased	Teixeira et al. (2012)

### 2.4 Contact-mediated ligands

#### 2.4.1 Cadherins and protocadherins

**FIGURE 7:**
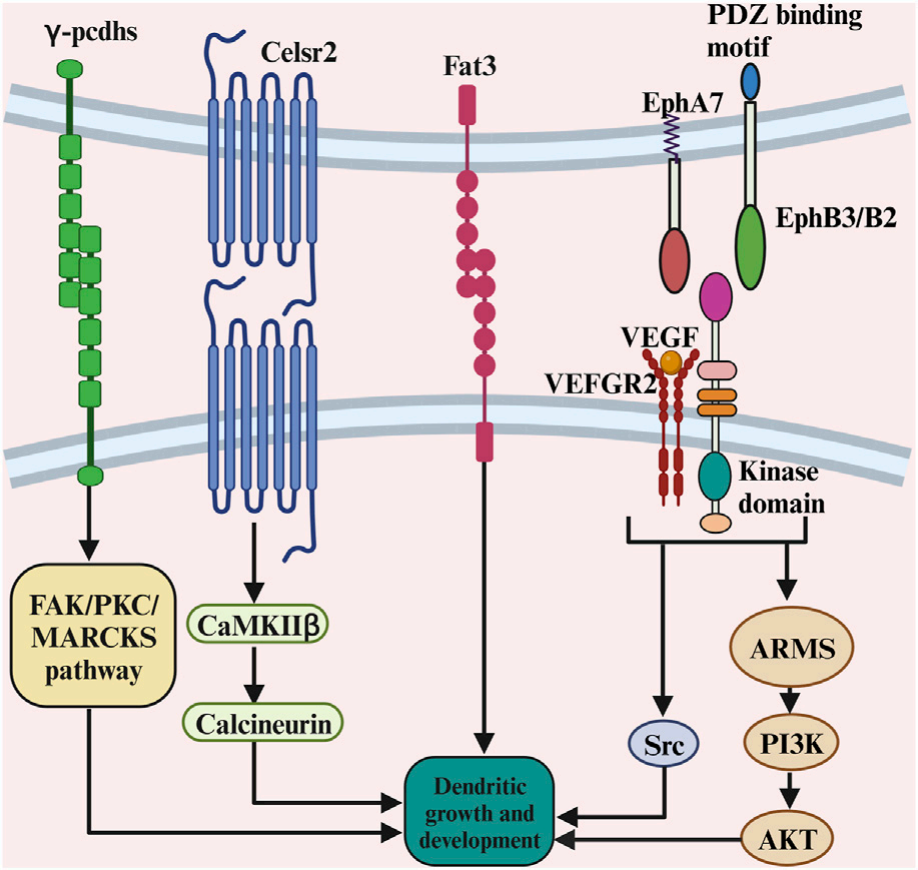
The complex role of contact-mediated ligands and its receptors in dendritic growth. The γ-protocadherins control cortical dendrite arborization by regulating the activity of a FAK/PKC/MARCKS signalling pathway. The effect of Celsr2 on dendritic growth depends on the activation of CaMKIIβ, which promotes dendritic growth. The ephrin-B3, functions postsynaptically as a receptor to transduce reverse signals into developing dendrites of mouse hippocampal neurons. The PI3K and Akt-mediated signalling pathway are crucial for ARMS-dependent dendrite development. There is a functional crosstalk of VEGFR2 and ephrinB2 *in vivo* to control dendritic growth, as VEGF activates the signalling partner of the Src family kinases to promote dendritic growth.

Cadherins are transmembrane proteins that mediate cell–cell adhesion. Cadherins play a crucial role in tissue morphogenesis and homeostasis by regulating contact formation and stability. Several cadherin/protocadherins have been implicated in dendrite growth. The studies on the role of cadherins and protocadherins in dendritic growth are summarised in Table 4. For instance, conditionally knocking out the γ-protocadherins (γ-pcdhs) in adult olfactory bulb granule cells decreased dendritic growth (Ledderose et al., 2013), as they control cortical dendrite arborization by regulating the activity of a FAK/PKC/MARCKS signalling pathway (Garrett et al., 2012; see also Figure 7). Eliminating Celsr2 seven-pass transmembrane cadherins decreased dendritic growth in neocortical pyramidal cells (Shima et al., 2007; see also Figure 7). The effect of Celsr2 on dendritic growth depends on the activation of CaMKIIβ, which promotes dendritic growth. Furthermore, eliminating Celsr3 seven-pass transmembrane cadherins in neocortical pyramidal neurons increased dendritic growth (Shima et al., 2007). The effect of Celsr3 on dendritic growth depends on calcineurin activation. Furthermore, γ-Pcdhs act locally to promote dendrite arborization via homophilic matching, and that depends on molecular interactions between neurons and between neurons and astrocytes (Molumby et al., 2016). Moreover, retinal amacrine cells (ACs) project primary dendrites into a discrete synaptic layer called the inner plexiform layer and rarely extend processes into other retinal layers. The atypical cadherin Fat3 ensures that ACs develop this unipolar morphology (Deans et al., 2011; see also Figure 7). AC precursors are initially multipolar but lose neurites as they migrate through the neuroblastic layer. In cadherin fat3 mutants, dendritic growth is enhanced (Deans et al., 2011; Krol et al., 2016). These studies suggest that cadherins and protocadherins are critical in dendritic development.

**TABLE 4:** Summary of the studies on the role of contact-mediated ligands and secreted and diffusible cues, and their receptors in dendritic growth. Days *in vitro* (DIV). Postnatal day (P).

Contact-mediated ligands					
1. Cadherins and protocadherins					
Protein	Animal and age	System	Strategy	Growth effect	Reference
**Celsr2**	mouse 10 DIV	cortical pyramidal neurons	knockdown	decreased	Shima et al. (2007)
**Celsr3**	mouse 10 DIV	cortical pyramidal neurons	knockdown	increased	Shima et al. (2007)
**cadherin Fat3**	mouse P1	retinal amacrine cells	knockout	increased	Deans et al. (2011)
**γ-pcdhs**	mouse 1 DIV	cortical neurons	mutation	increased	Garrett et al. (2012)
**γ-pcdhs**	mouse adult	olfactory bulb granule cell	knockout	decreased	Ledderose et al. (2013)
**2. Ephrins**					
**EphA7**	mouse P10	cortical pyramidal cells	knockout	increased	Clifford et al. (2014)
**EphB2**	rat 13 DIV	hippocampal neurons	overexpression	increased	Hoogenraad et al. (2005)
**EphB2**	mouse P10	hippocampal neurons	knockout	decreased	Harde et al. (2019)
**EphB3**	mouse P9-20	Pyramidal neurons	knockout	increased	Xu et al. (2011)
**Secreted and diffusible cues**					
**1. Wingless (Wnts)**					
**Fz7**	rat P7	hippocampal granule cells	knockdown	decreased	Ferrari et al. (2018)
**Ryk**	mouse P14	Dissociated hippocampal and cortical neuronal	knockdown	increased	Lanoue et al. (2017)
**Wnt7b**	mouse 9 DIV	cultured hippocampal neurons	activation	increased	Rosso et al. (2005)
**Wnt-2**	mouse 7 DIV	hippocampal neurons	overexpression	increased	Wayman et al. (2006)
**Wnt5a**	mouse P5	olfactory bulb interneurons	knockout	decreased	Pino et al. (2011)
**Wnt7b**	mouse 9 DIV	cultured hippocampal neurons	activation	increased	Rosso et al. (2005)
**2. Semaphorins**					
**Neuropilins 1 or 2**	mouse adult	dentate gyrus neurons	knockdown	decreased	Ng et al. (2013)
**PlexinA3**	rat 5 DIV	cultured cerebellar granule neurons	knockdown	decreased	Jiang et al. (2020)
**PlexinB1**	mouse 7 DIV	cortical neurons	activation	decreased	Saito et al. (2009)
**Sema3A**	mouse P7	cortical pyramidal cells	knockout	decreased	Fenstermaker et al. (2004)
**Sema3A**	mouse 1–3 months	hippocampal CA1 pyramidal neurons	knockout	increased	Nakamura et al. (2009)
**Sema3A**	mouse 4 DIV	hippocampal neurons	activation	increased	Schlomann et al. (2009)
**Sema3A**	mouse	cultured cortical neuron	activation	increased	Kawashima et al. (2021)
**Sema3A**	mouse 4–5 DIV	cortical neuron	activation	increased	Danelon et al. (2020)
**3. Neuregulins**					
**Neuregulin**	rat 15 DIV	retinal cell cultures	activation	increased	Bermingham-McDonogh et al. (1996)
**Neuregulin**	rat 6 DIV	cerebellar granule cells	activation	increased	Rieff et al. (1999)
**Nrg-1, -2 and -3**	rat 2 DIV	cortical interneurons	activation	increased	Rahman-Enyart et al. (2020)
**Nrg-1beta**	rat 4 DIV	cultured hippocampal neurons	activation	increased	Gerecke et al. (2004)
**Nrg-4**	mouse 10–12 DIV	striatal medium spiny neurons	knockout	increased	Paramo et al. (2019)
**Nrg-4**	mouse P10	cortical pyramidal cells	knockout	decreased	Paramo et al. (2021)

#### 2.4.2 Ephrins

The Eph receptors represent mammals largest known family of receptor tyrosine kinases. These receptors are critical for a variety of normal cellular processes during development. The studies on the role of ephrins in dendritic growth are summarised in Table 4. It has been shown that hippocampal neurons showed a reduced dendritic size in EphB receptor (EphBR) knockout mice (Hoogenraad et al., 2005). A possible explanation is that the extracellular domain of EphBR is involved in the clustering and functional enhancement of NMDA receptor expression, which plays a role in dendritic development (Grunwald et al., 2001). Moreover, cortical pyramidal cells showed increased dendritic size in EphA7 knockout mice compared to wild-type mice (Clifford et al., 2014). Furthermore, the ephrin-B3, a transmembrane ligand for Eph receptors, functions postsynaptically as a receptor to transduce reverse signals into developing dendrites of mouse hippocampal neurons (Figure 7). Both tyrosine phosphorylation-dependent GRB4 SH2/SH3 adaptor-mediated signals and PSD-95-discs large-zona occludens-1 (PDZ) domain-dependent signals are required for inhibition of dendrite branching (Xu et al., 2011). Furthermore, suppressing ankyrin repeat-rich membrane-spanning protein (ARMS) using *in utero* electroporation reduced cortical neurons dendritic growth, as ankyrin is required for neurotrophin and ephrin receptor-dependent dendrite development (Chen et al., 2012). The PI3K and Akt-mediated signalling pathways are crucial for ARMS-dependent dendrite development (Figure 7). Recently, it has been shown that there is a functional crosstalk of VEGFR2 and ephrinB2 *in vivo* to control dendritic arborization (Harde et al., 2019). Hippocampal pyramidal cells showed reduced dendritic growth in the vascular endothelial growth factor receptor 2 (VEGFR2) knockout mice (Harde et al., 2019), as VEGF activates the signalling partner of the Src family kinases to promote dendritic growth (Figure 7). Taken together, these studies suggest the role of ephrins in dendritic growth.

### 2.5 Secreted and diffusible cues

#### 2.5.1 Wingless (Wnts)

**FIGURE 8:**
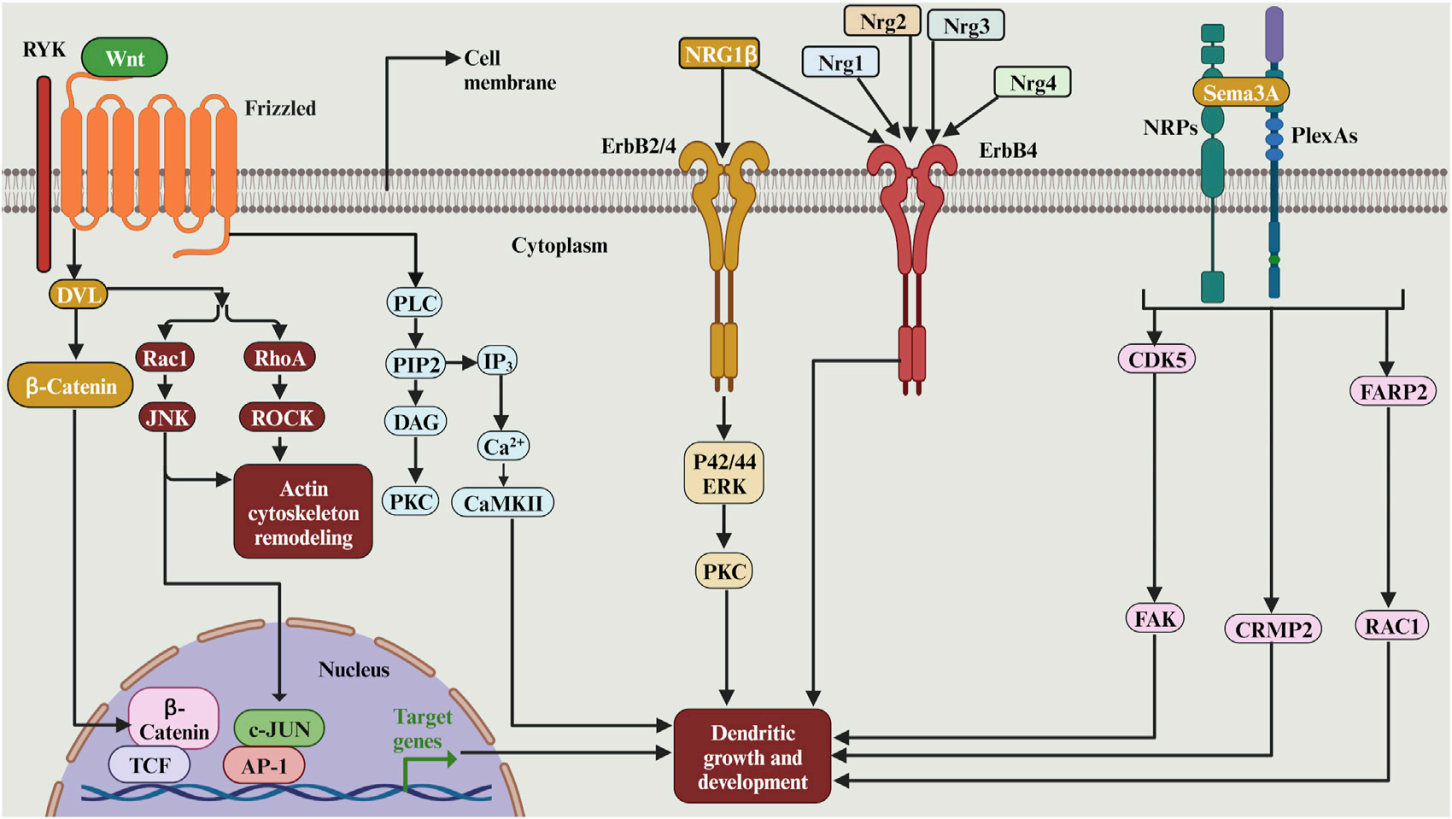
The complex role of secreted and diffusible cues and its receptors in dendritic growth. WNTs can signal through the tyrosine kinase-related receptor RYK, which interacts with FZ and DVL. Downstream of DVL, the WNT pathway diverges into at least three branches: the canonical or WNT/β-catenin pathway, the planar cell polarity (PCP) pathway and the WNT/Ca^2+^ pathway. The Sema3A facilitate the initial growth of CA1 apical dendrites via the activation of p35/Cdk5, which may in turn signal hippocampal development. The Sema3A regulates basal pyramidal dendritic length on cortical neurons via Sema3A-Nrp1/PlxnA4/FARP2/Rac1 signalling pathway that specifically controls dendritic morphogenesis. The neuregulin NRG-1beta stimulates hippocampal dendrite extension and arborization via a signalling pathway that involves erbB membrane tyrosine kinases (erbB2 and/or erbB4), p42/44 ERK, and PKC. The Nrg4 binds to its receptor ErbB4 and promotes dendritic growth of the striatal medium spiny neurons. In cortical GABAergic inhibitory interneurons, the neuregulins 1, 2, and 3 promote early dendritic growth in ErbB4-expressing cells.

Wingless/Wnts are signalling molecules which play a role in dendritic development. WNTs signal through their Frizzled (FZ) receptors to be able to activate the cytoplasmic scaffolding protein Dishevelled (DVL). WNTs can also signal through the tyrosine kinase-related receptor RYK, which interacts with FZ and DVL (Figure 8). Downstream of DVL, the WNT pathway diverges into at least three branches: the canonical or WNT/β-catenin pathway (Figure 8), the planar cell polarity (PCP) pathway and the WNT/Ca^2+^ pathway (Ciani and Salinas, 2005). The studies on the role of Wnts in dendritic growth are summarised in Table 4. Eliminating the Wnt Ryk receptor in dissociated hippocampal and cortical neurons also increased dendritic growth (Lanoue et al., 2017). Ryk is known to signal through the PCP pathway, and the depletion of Ryk expands the dendritic tree. Furthermore, when overexpressed, the Wnt5a-Frizzled4 signalling pathway has increased the dendritic growth of the developing hippocampal neurons. The growth effect of Fzd4 was mediated by the downstream signalling cascade of the distal PDZ-binding motif of Fzd4 (Bian et al., 2015). The elimination of Frizzled-7 (Fz7) reduced the dendritic growth of granule cells (Ferrari et al., 2018). The Fz7 induces the phosphorylation of CaMKIIβ, which mediates dendritic growth. Furthermore, Wnt7b, expressed in the mouse hippocampus, increases dendritic branching in cultured hippocampal neurons through Dishevelled, Rac and JNK (Rosso et al., 2005). Moreover, Wnt-2 increased dendritic growth and branching of primary hippocampal neurons (Wayman et al., 2006). Finally, the non-canonical Wnt pathway induced by Wnt5a is important for the morphological development of olfactory bulb interneurons both *in vitro* and *in vivo* (Pino et al., 2011). Together. these studies suggest that the Wnt signalling molecules are important for dendritic development.

#### 2.5.2 Semaphorins

Semaphorins comprise a large family of conserved proteins that act as guidance cues and dendritic development and guidance in many organisms. The studies on the role of semaphorins in dendritic growth are summarised in (Table 4). Semaphorin 3A (Sema3A), a class III semaphorin, has been shown to regulate the radial orientation of pyramidal neurons in the developing neocortex (Figure 8). It is implicated in the elaborating second and third-order dendritic branches in pyramidal neurons in the neocortex (Fenstermaker et al., 2004). In Sema3A mutant mice, atypical proximal bifurcation of CA1 pyramidal apical dendrites was increased (Nakamura et al., 2009). The proximal bifurcation of CA1 pyramidal neurons was also increased in the mutant mice of p35, an activator of cyclin-dependent kinase 5 (Cdk5), suggesting that Sema3A may facilitate the initial growth of CA1 apical dendrites via the activation of p35/Cdk5, which may in turn signal hippocampal development (Nakamura et al., 2009). Interestingly, the Sema3A controls cortical development through remote signalling. It has been proposed that retrograde Sema3A signalling regulates the glutamate receptor localization through the trafficking of AMPAR GluA2 along dendrites; this remote signalling may be an alternative mechanism to local adhesive contacts for neural network formation (Goshima et al., 2016). Moreover, the neutralisation of plexin-B1, the semaphorin 4D (Sema4D) receptor, with an antibody resulted in a reduction in the dendritic growth of dissociated neocortical neurons (Saito et al., 2009) since Plexin B1, which is a dual functional GAP for R-Ras and M-Ras, remodels dendrite morphology. Moreover, silencing of semaphorin receptors neuropilins (NRP) 1 or 2 in neural progenitors at the adult mouse dentate gyrus resulted in newly differentiated neurons with shorter dendrites and simpler branching *in vivo* through the activation of Cdk5-FAK signalling pathway (Ng et al., 2013). Moreover, Sema3A promotes the extension of hippocampal dendrites by a pathway that requires focal adhesion kinase (FAK). The stimulation of dendrite growth and FAK phosphorylation by Sema3A depends on integrin engagement (Schlomann et al., 2009). Moreover, the Sema3A-regulated dendritic development of cortical pyramidal neurons required phosphorylation of collapsin response mediator proteins (CRMP1) by Fyn is an essential step (Kawashima et al., 2021). Furthermore, Sema3A regulates basal pyramidal dendritic length on cortical neurons via Sema3A-Nrp1/PlxnA4/FARP2/Rac1 signalling pathway that specifically controls dendritic morphogenesis (Danelon et al., 2020; see also Figure 8). Moreover, Sema3A signalling during dendritic growth in cerebellar granule neurons requires the PlexinA3/CRMP2 pathway (Jiang et al., 2020, see also Figure 8). Taken together, these studies suggest the role of the Sema3A in modulating dendritic architecture in early development.

#### 2.5.3 Neuregulins

Neuregulins are a family of four structurally related proteins making part of the EGF family. These neuregulins have been shown to have multiple functions during the development of the nervous system. Many studies have shown that neuregulins can regulate dendritic development. The studies on the role of neuregulins in dendritic growth are summarised in (Table 4). For instance, in rat retinal cell cultures, when added to cultures of embryonic or neonatal rat retinal cells, neuregulin (rhGGF2) promotes survival and neurite extension from retinal neurons in a dose-dependent manner (Bermingham-McDonogh et al., 1996). Moreover, the neuregulin Ig-NRG plays a crucial role in cerebellar granule cell dendrite outgrowth by increasing the expression of the GABA_A_R beta2 subunit (Rieff et al., 1999). Furthermore, it has been shown that the neuregulin NRG-1beta stimulates hippocampal dendrite extension and arborization via a signalling pathway that involves erbB membrane tyrosine kinases (erbB2 and/or erbB4), p42/44 ERK, and PKC (Gerecke et al., 2004; see also Figure 8). Moreover, it has been shown that neuregulin-4 (Nrg4) binds to its receptor ErbB4 and promotes dendritic growth of the striatal medium spiny neurons (Paramo et al., 2019). Indeed, in the motor cortex, the Nrg4 is required to maintain the pyramidal cells soma size (Paramo et al., 2021). Finally, in cortical GABAergic inhibitory interneurons, the neuregulins 1, 2, and 3 promote early dendritic growth in ErbB4-expressing cells (Rahman-Enyart et al., 2020; see also Figure 8). Thus, the neuregulin plays a key role in early dendritic development.

## 3 Conclusion and perspectives

Over the past 3 decades, studies of dendrite development and its associated signalling cascade mechanisms that regulate dendrite outgrowth have enormously expanded. We have focused in this review on receptor control of dendrite development. Most of these studies have been performed in an early developmental time window because dendrites in early development undergo massive growth and retraction. In early development, the neuronal network starts to mature and become spontaneously active, which coincides with the period of rapid dendritic growth dependent on Ca^2+^ signalling (Garaschuk et al., 2000; Ben-Ari, 2001). Moreover, in the early developmental stage, neurons start to express a subset of receptors to facilitate neurone-neurone contact and to promote the activation of a certain signal cascade, which may be an important signal for neuronal differentiation. The outcome of the overexpression of receptors studies has been inconclusive. Some studies reported increased dendritic growth, while others reported a suppression in dendritic growth. Ca^2+^ permeability is a crucial factor in promoting dendritic growth. In fact, most of the overexpressed Ca^2+^-impermeable glutamate receptors did not alter dendritic morphology, indicating that Ca^2+^ signalling is indispensable for dendritic development. Depending on the activated signal cascade downstream of the receptor, the dendrite might grow or stop growing. Thus, overexpressing or activating a certain receptor is not guaranteed for neurons to grow unless that receptor is coupled to a growth-promoting signal cascade.

Similarly, eliminating or inhibiting certain receptors does not imply that the cell will stop growing unless that receptor is coupled to a growth-promoting signal cascade. Neuronal hypotrophy will not allow neurons to establish and receive enough contact from neighbouring cells, resulting in poor synaptic transmission. On the other hand, hypertrophic dendrites are also pathological. Neuronal hypertrophy can lead to pathological hyperexcitability of neuronal circuits, which can contribute to disorders such as epilepsy, schizophrenia or bipolar disorder, autism spectrum disorder (Courchesne and Pierce, 2005; Ishii et al., 2016; Hu et al., 2017). Therefore, it is necessary to have ‘stop growth signals’ to transform dynamic, immature dendrites into more stable mature dendrites and establish balanced neuronal network connectivity. Any disruption of the dendritic development process can contribute to neurological disorders associated with cognitive deficits. Exploring the mechanisms that regulate dendritic growth will pave the way for future studies to improve understanding of neurological diseases.
